# Supplementary material for: Towards synthetic PETtrophy: Engineering Pseudomonas putida for concurrent polyethylene terephthalate (PET) monomer metabolism and PET hydrolase expression
Source: Microb Cell Fact. 2022 Jun 18;21:119. doi: 10.1186/s12934-022-01849-7 (PMC9206389; doi:10.1186/s12934-022-01849-7)
Supplement: Supplementary file 1 — Additional file 1. Figure S1: Design of pBAMD-tphII. Figure S2: ALE with P. putida TA for growth at elevated pH. Figure S3: Rescue of P. putida TA growth at elevated pH by plasmid-based mhpT overexpression. Figure S4: Negative effect of high EG concentrations on P. putida growth, and the P. putida EG metabolic pathway. Figure S5: ALE with P. putida TA7 for EG utilization. Figure S6: Cell dry weight measurements of P. putida TA7 and TA7-EG. Figure S7: Growth comparisons of P. putida TA7, TA7-EG, and TA7-BD in LB and M9 minimal media. Figure S8: ALE with P. putida TA7 for BD utilization. Figure S9: Design of PET hydrolase membrane display expression vectors. Figure S10: pNPB assay establishment. Figure S11: SDS-PAGE analysis of EhaA and OprF membrane display constructs. Figure S12: Plate reader analysis of EhaA and OprF PET hydrolase membrane display construct expression growth curves. Figure S13: Plate reader analysis of LCC, HiC, and IsP cytosolic expression. Figure S14: Rhamnose inducer titration with pLo- PrhaB-EhaA-LCC. Figure S15: Plate reader analysis of PET hydrolase secretion construct expression growth curves. Figure S16: Assessing HiC esterase activity in cell culture supernatant, whole-cell fraction, and cell lysate. Figure S17: Comparing growth of P. putida TA7-EG transformed with different promoter system empty vector plasmids. Figure S18: SDS-PAGE analysis of PIbpA-bglx-HiC and PIbpA-lasb-IsPDura secretion constructs. Figure S19: Comparing EhaA-LCC expression output from different promoter systems. Figure S20: Testing for aPET plastic degradation with P. putida KT2440 PIbpA-bglx-HiC. Table S1: Genomic landing sites of knock-in cassettes. Table S2: Whole-genome sequencing of engineered P. putida strains. Table S3: Signal peptide oligonucleotide library. Table S4: SP library screening data. Table S5: Plasmids used in this work. Table S6: Bacterial strains used in this work. Table S7: Oligonucleotides used in this work. Sequence S1. Sequence S2. Sequence S [file 12934_2022_1849_MOESM1_ESM.pdf]

Supplementary Information for:

Towards synthetic PETtrophy: Engineering *Pseudomonas putida* for concurrent polyethylene terephthalate (PET) monomer metabolism and PET hydrolase expression

Oliver F. Brandenberg[1,*,✉], Olga T. Schubert[1,2,3,*], Leonid Kruglyak[1,✉]

[1] Department of Human Genetics, Department of Biological Chemistry, and Howard Hughes Medical Institute, University of California Los Angeles, Los Angeles, United States.

[2] Department of Environmental Microbiology, EAWAG, 8600 Dübendorf, Switzerland.

[3] Department of Environmental Systems Science, ETH Zurich, 8092 Zürich, Switzerland.

[*] Equal contribution

✉ Corresponding authors: Oliver F. Brandenberg brandenberg.oliver@gmail.com

Leonid Kruglyak lkruglyak@mednet.ucla.edu

Contents:

I	Supplementary Figures	p. 2
II	Supplementary Tables	p. 14
III	Supplementary DNA Sequences	p. 27
Iv	Supplementary References	p. 36

I Supplementary Figures

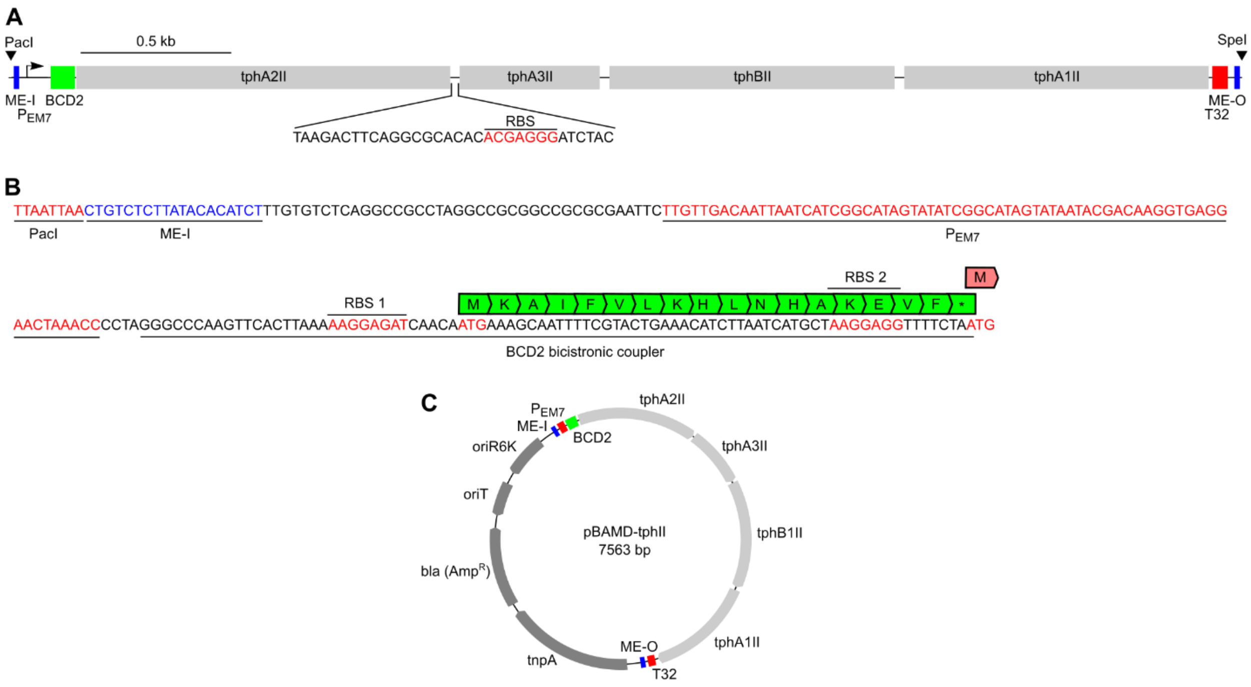

Figure S1: Design of pBAMD-tphII. (A) Layout of the tph_{II} synthetic operon. The knock-in cassette is flanked by unique PacI and SpeI restriction sites, followed by the ME-I and ME-O 5' and 3' end transposase recognition sites, respectively. The operon is under transcriptional control of the constitutive P_{EM7} promoter and endowed with a BCD2 bicistronic translational coupler. The four ORFs are separated by 32 bp intergenic regions, each containing an additional ribosome binding site. The operon is followed by a T32 transcriptional terminator. (B) Sequence view of the knock-in cassette 5' end from the PacI site to the ATG of *tphA2*$_{II}$, showing ME-I (blue), P_{EM7} (red), and the BCD2 translational coupler including the amino acid sequence of its short ORF. (C) Plasmid map of pBAMD-tphII; all functional elements outside of the tph_{II} operon remained unchanged and as described for the original pBAMD1-2 plasmid.[1]

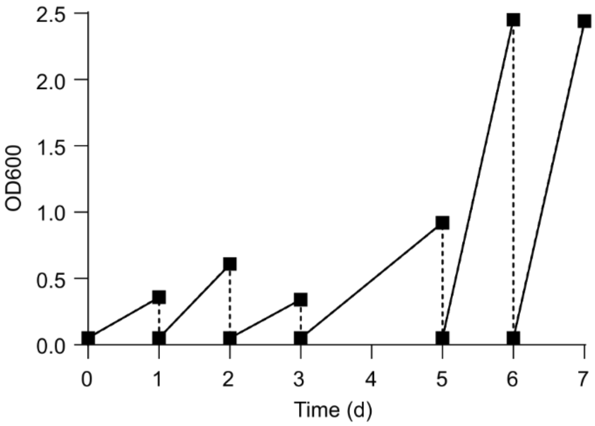

Figure S2: ALE with *P. putida* TA for growth at elevated pH. *P. putida* TA was cultured in M9 minimal medium, 0.4% (w/v) TA, pH 6.5, for 7 days (6 passages). After 7 days we observed improved bacterial growth and proceeded to the isolation and analysis of single clones, which yielded strain *P. putida* TA7.

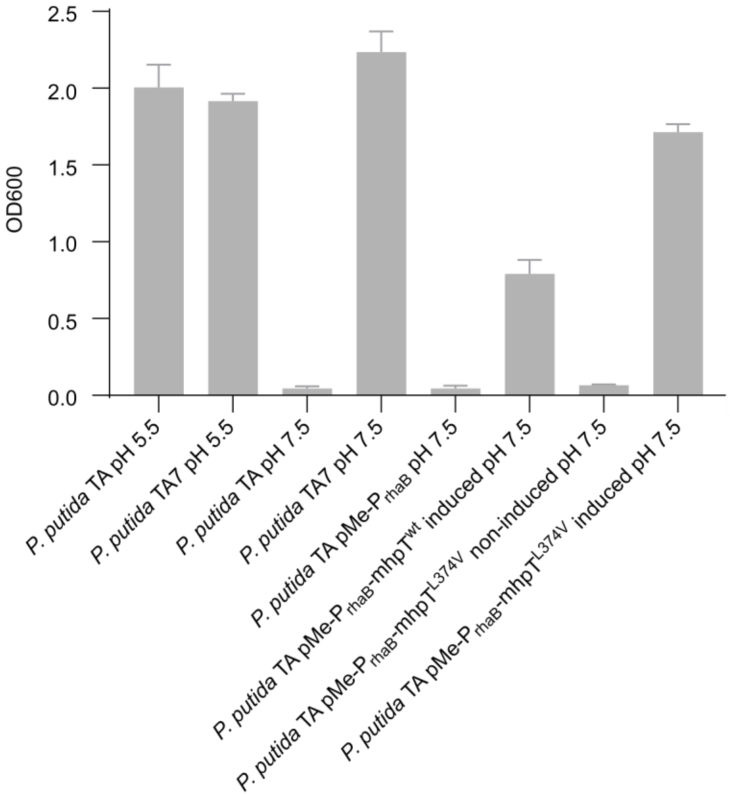

Figure S3: Rescue of *P. putida* TA growth at elevated pH by plasmid-based *mhpT* overexpression. While both *P. putida* TA and TA7 grow well in M9 minimal medium with 0.4% TA at pH 5.5, only *P. putida* TA7 grows at pH 7.5. However, growth of *P. putida* TA at pH 7.5 can be rescued, to some extent, by plasmid-based overexpression of *mhpT*^{wt} (from a medium copy number plasmid and under control of the P_{rhaB} promoter), indicating promiscuous function of MhpT as TA transporter. Better rescue of *P. putida* TA

at pH 7.5, approaching the growth level of *P. putida* TA7, can be achieved by overexpression of *mhpT*^{L374V}, while no rescue is seen for e.v. or non-induced controls. Data shown are mean and S.D., n=2.

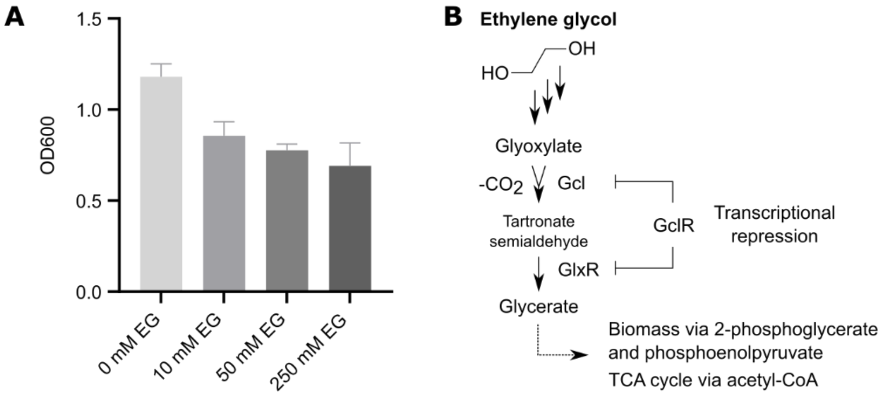

Figure S4: Negative effect of high EG concentrations on *P. putida* growth, and the *P. putida* EG metabolic pathway. (A) Growth (OD600) of *P. putida* TA7 overnight cultures in M9 minimal medium, 10 mM TA, pH 7.5, with varying amounts of EG; shown are mean and S.D from n=2 cultures. (B) Pathway of EG metabolization in *P. putida*, showing the crucial function of the GclR transcriptional repressor, blocking expression of the enzymes Gcl and GlxR required for productive EG metabolism.[2–4]

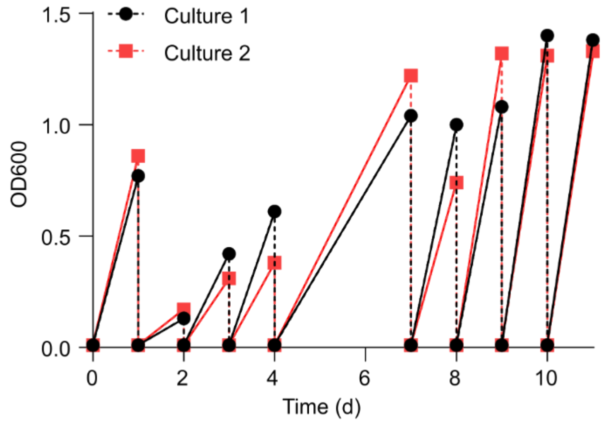

Figure S5: ALE with *P. putida* TA7 for EG utilization. We performed ALE with *P. putida* TA7 in duplicate cultures using M9 minimal medium containing 25 mM EG and 5 mM TA, pH 7.5. Single clones were isolated on day 11 followed by further characterization and whole-genome sequencing, yielding strain *P. putida* TA7-EG. This strain carries a likely *gclR* loss-of-function mutation (c.388G>T, E130*).

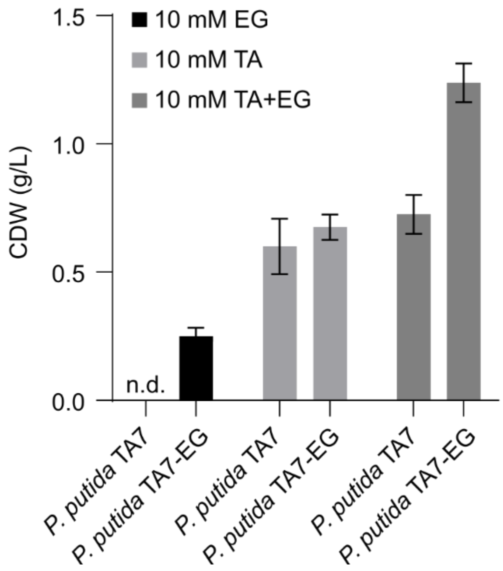

Figure S6: Cell dry weight measurements of *P. putida* TA7 and TA7-EG. Both strains were cultured for 24 h in M9 minimal medium, pH 7.5, with the indicated PET monomer concentrations. Mean and S.D. of duplicate cultures are shown.

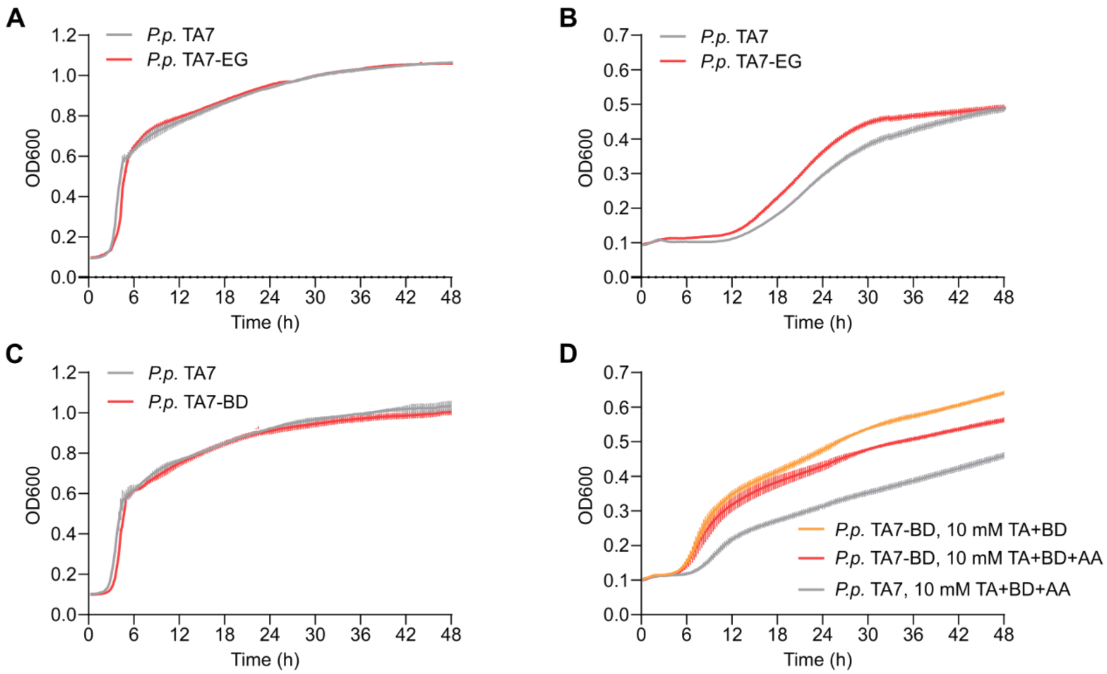

Figure S7: Growth comparisons of *P. putida* TA7, TA7-EG, and TA7-BD in LB and M9 minimal media. (A) Growth comparison of *P. putida* TA7 and TA7-EG in LB medium. (B) Growth comparison of *P. putida* TA7 and TA7-EG in M9 minimal medium, 10 mM TA, pH 7.5. Despite not showing any mutations in the *tph*$_{II}$ cassette, strain TA7-EG showed a slight growth advantage over strain TA7 with TA as sole carbon source. (C) Growth comparison of *P. putida* TA7 and TA7-BD in LB medium. (D) Growth comparison of *P. putida*

TA7 and TA7-BD in M9 minimal medium, 10 mM TA, 10 mM BD, and with / without 10 mM AA, pH 7.5. Data shown are mean and S.D. from n=6 wells per strain.

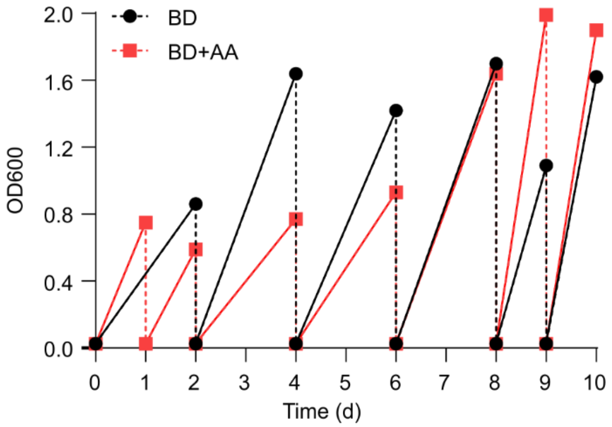

Figure S8: ALE with *P. putida* TA7 for BD utilization. We performed ALE with *P. putida* TA7 in two cultures using M9 minimal medium containing either 25 mM BD and 5 mM TA, or 25 mM BD, 5 mM TA, and 25 mM AA. Single clones were isolated on day 10 followed by further characterization and whole-genome sequencing, yielding strain *P. putida* TA7-BD. This strain carries a mutation in the LysR-family transcriptional regulator PP_2046 (c.407T>C, V136A) likely influencing BD metabolism.[5]

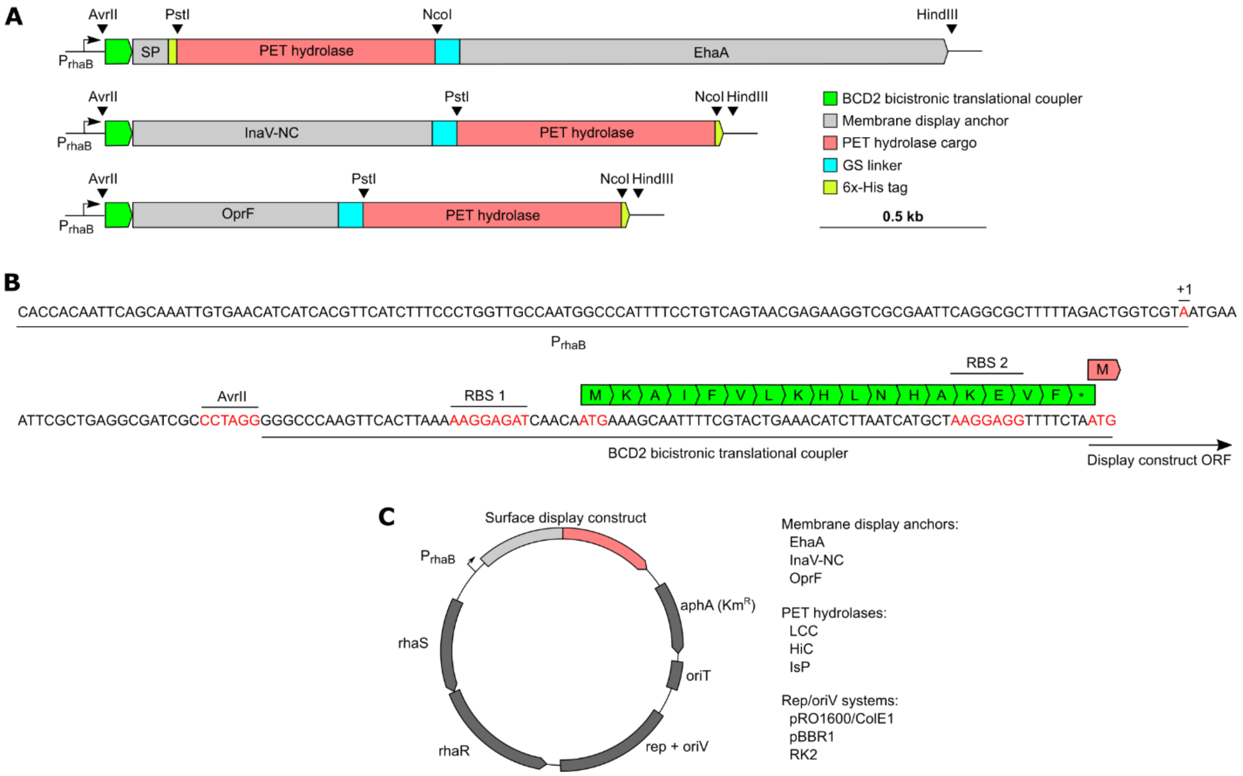

Figure S9: Design of PET hydrolase membrane display expression vectors. (A) Overview of membrane display expression cassettes, including EhaA (top), InaV-NC (middle), and OprF (bottom). The display cassettes are flanked by unique AvrII and HindIII restriction sites, are under transcriptional control of the P_{rhaB} promoter, and contain unique PstI and NcoI sites to easily clone cargo domains (here: PET hydrolases) into each display anchor system. Note that the cargo domain is positioned N-terminally to the display anchor domain in EhaA, while the cargo sits C-terminally in both InaV-NC and OprF. In all three constructs, cargo and display anchor are connected via a $G_4SGGS(G_4S)_3$ linker, and the PET hydrolase cargos contain a 6x-His tag (N-terminal in EhaA constructs, C-terminal in InaV-NC and OprF constructs). The PstI site was embedded in a 9 bp sequence (cctgcaggc) to maintain mostly neutral amino acids (PAG); likewise, the NcoI site was embedded in a 9 bp sequence (ggccatggg, GHG). (B) Sequence view of the P_{rhaB} promoter-BCD2 region, containing P_{rhaB}, AvrII restriction site, BCD2 bicistronic translational coupler, and the ATG of the display construct. (C) Generalized plasmid map of the utilized membrane display vectors; we used three vector systems differing in their replication system, providing low (RK2), medium (pBBR1), and high (pRO1600/ColE1) cellular copy numbers, in combination with the three membrane display anchors and the three PET hydrolases.

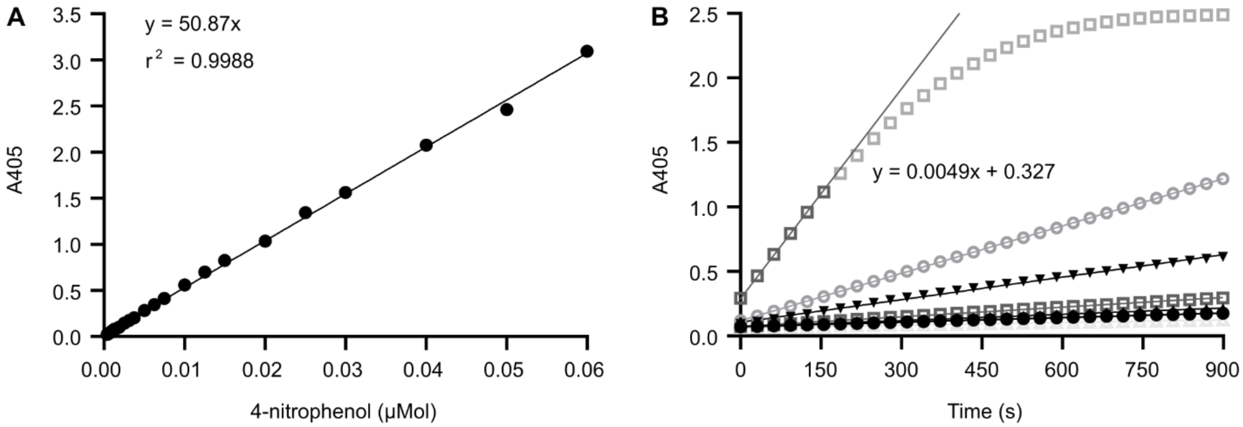

Figure S10: pNPB assay establishment. (A) Calibration curve for 4-nitrophenol (4NP; see Methods for details). (B) Exemplary experimental data. 4NP formation from pNPB was measured in 96-well plates over 15 min (measurement intervals: 31 s) in a plate reader. Only those data points falling into the linear range of the assay were used to derive the slopes of the curves using linear regression. As an example, the datapoints of the topmost curve used for the calculation (darker grey) together with the resulting equation are indicated. The slope was subsequently used to derive 4NP formation over time using the slope of the calibration curve.

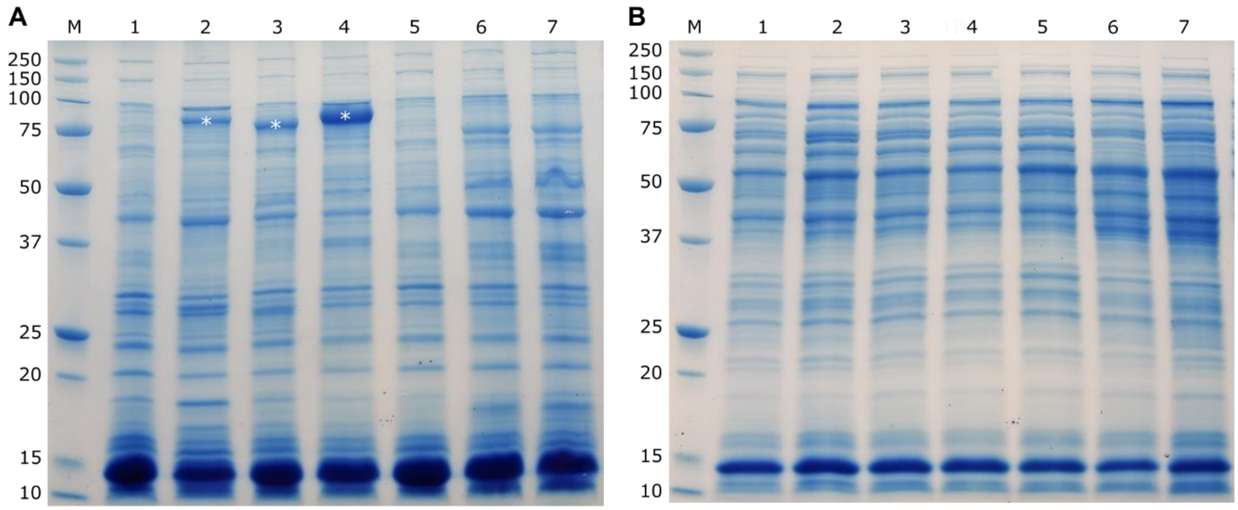

Figure S11: SDS-PAGE analysis of EhaA and OprF membrane display constructs. (A) SDS-PAGE of the membrane fraction of cells expressing PET hydrolase display constructs. M, marker; 1 pLo-P_{rhaB}; 2 pLo-P_{rhaB}-EhaA-LCC (85.2 kDa); 3 pLo-P_{rhaB}-EhaA-HiC (77.6 kDa), 4 pLo-P_{rhaB}-EhaA-IsP (84.8 kDa), 5 pLo-P_{rhaB}-OprF-LCC (50.3 kDa), 6 pLo-P_{rhaB}-OprF-HiC (42.6 kDa), 7 pLo-P_{rhaB}-OprF-IsP (50.0 kDa). For calculating the shown theoretical molecular weights, we assumed that the OprF SP (present in both EhaA and OprF constructs) is cleaved off at position 24 (signal peptidase recognition site AMA). Asterisks mark the putative bands belonging to the EhaA expression constructs, which were excised from the gel, and their

identity confirmed by mass spectrometry. (B) SDS-PAGE of the cytosolic fraction of cells expressing PET hydrolase display constructs; loading as in (A). No substantial accumulation of the display constructs in the cytosolic fraction was observed.

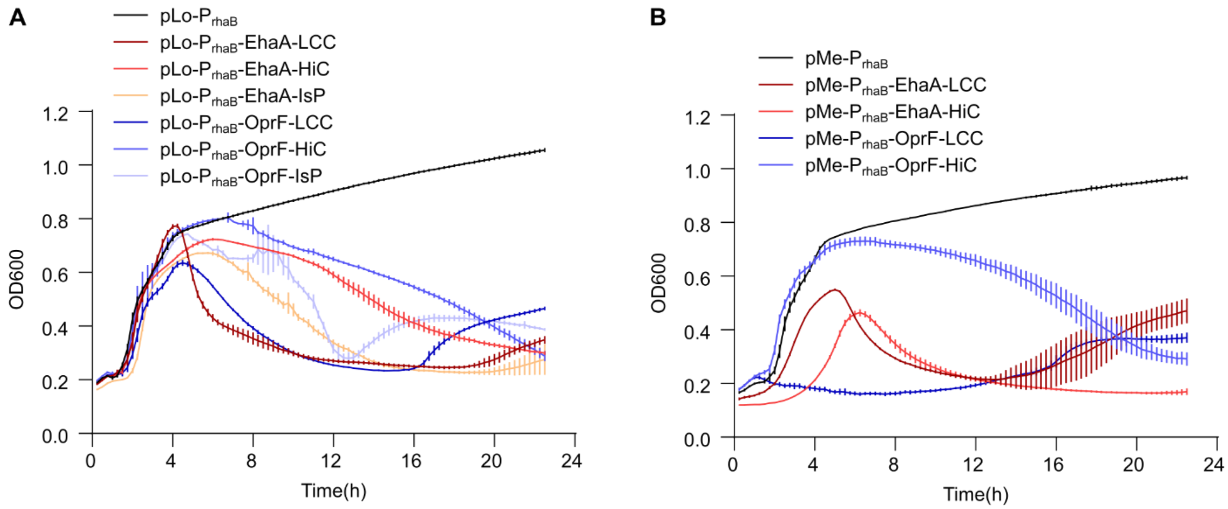

Figure S12: Plate reader analysis of EhaA and OprF PET hydrolase membrane display construct expression growth curves. Cultures of *P. putida* TA7-EG transformed with the indicated plasmids were grown in LB medium in 96-well plates and OD600 was measured in 15 min intervals for 24 h post-induction (induction with 2 mM rhamnose at t=0 h). (A) Membrane display constructs expressed from low copy number vectors (RK2). (B) Membrane display constructs expressed from medium copy number vectors (pBBR1). Mean and S.D. from a representative experiment with n=6 wells per construct are shown.

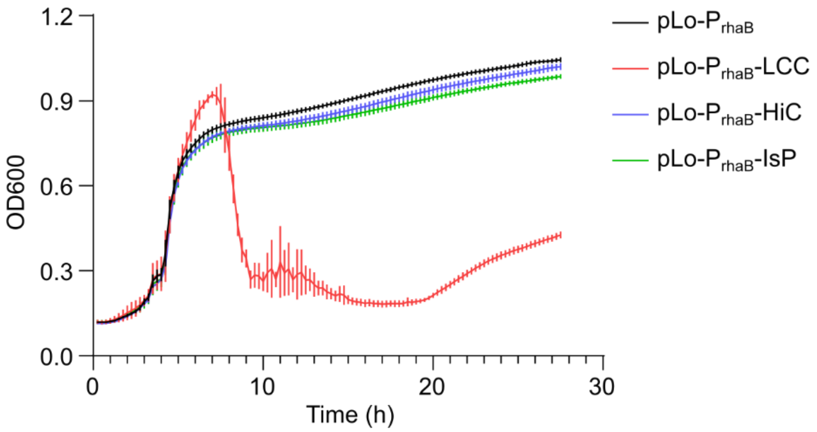

Figure S13: Plate reader analysis of LCC, HiC, and IsP cytosolic expression. *P. putida* TA7-EG cultures expressing cytosolic constructs of the three PET hydrolases were grown in 96-well plates and

OD600 was measured in 15 min intervals for 24 h post-induction (induction at t=0 h). Mean and S.D. from a representative experiment with n=10 wells per construct are shown.

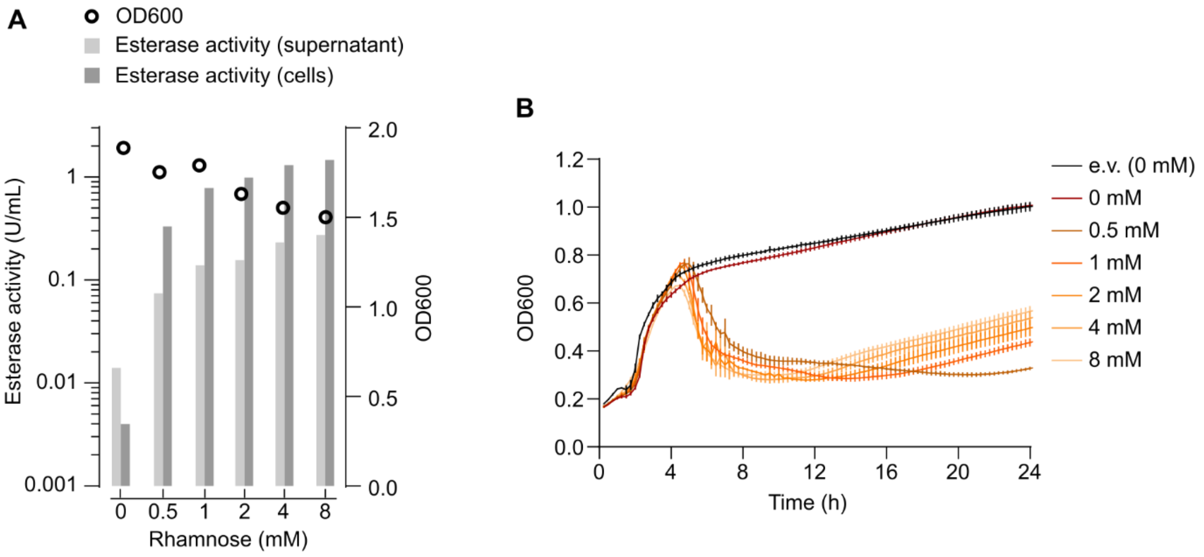

Figure S14: Rhamnose inducer titration with pLo-P$_{rhaB}$-EhaA-LCC. (A) *P. putida* TA7-EG cultures transformed with pLo-P$_{rhaB}$-EhaA-LCC were induced with the indicated amounts of rhamnose, and OD600 and esterase activity were measured 3 h post-induction (before onset of culture collapse). (B) Plate reader analysis of the same expression culture setup (and including pLo-P$_{rhaB}$ as e.v. control) induced with the indicated amounts of rhamnose; culture OD600 was measured for 24 h post-induction (induction at t=0 h). Data in (A) are derived from a single representative experiment performed with duplicate cultures, while data shown in (B) are derived from a single representative experiment with n=6 wells per strain.

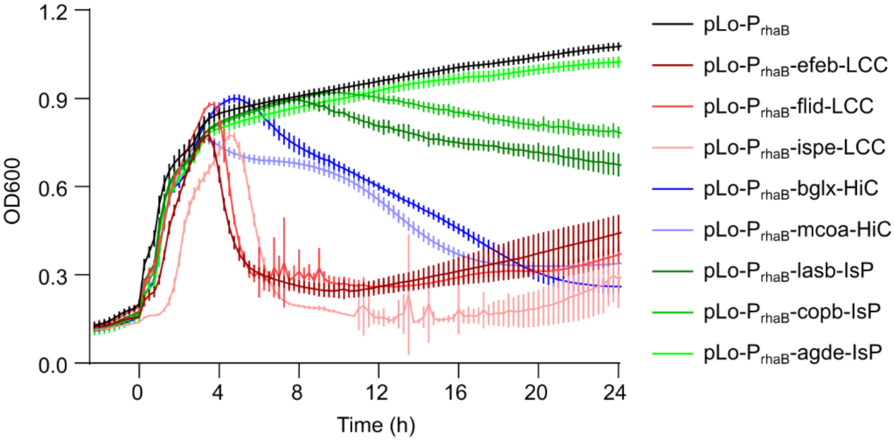

Figure S15: Plate reader analysis of PET hydrolase secretion construct expression growth curves. *P. putida* TA7-EG cultures transformed with the indicated plasmids were grown in LB medium in 96-well

plates and OD600 was measured in 15 min intervals for 24 h post-induction (induction at t=0 h). Mean and S.D. from a representative experiment with n=6 wells per construct are shown.

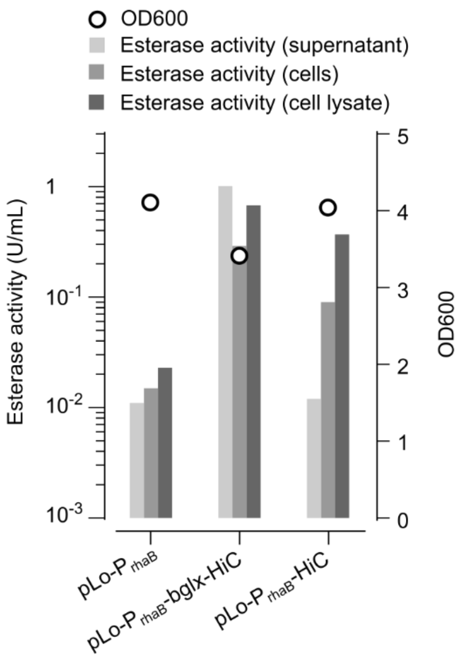

Figure S16: Assessing HiC esterase activity in cell culture supernatant, whole-cell fraction, and cell lysate. *P. putida* TA7-EG cultures transformed with the indicated plasmids were cultured for 3 h post-induction, and esterase activity in the supernatant, cell fraction, and cell lysate was assessed. We assume that the strong cell-associated esterase signal for the secreted pLo-P_{rhaB}-bglx-HiC construct originates from the PET hydrolase *en route* to secretion but still in the cell's periplasm, where it may have access to the pNPB substrate through outer membrane porins. Cytosolic expression does not result in apparent HiC accumulation in the supernatant and a lower cell-associated esterase signal.

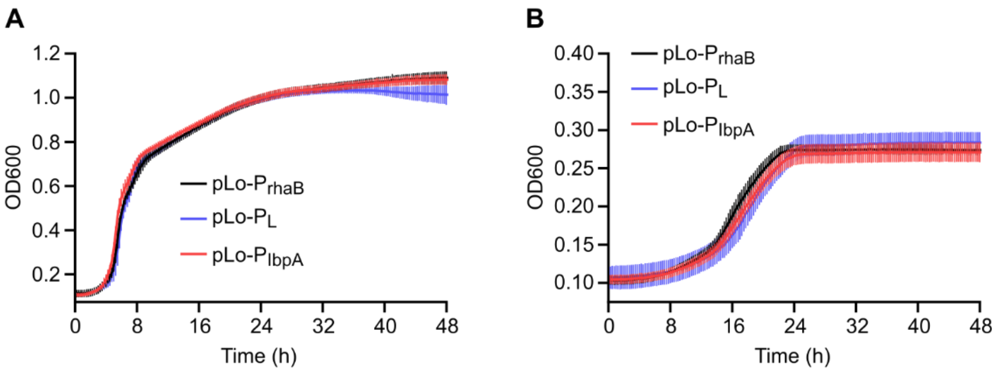

Figure S17: Comparing growth of *P. putida* TA7-EG transformed with different promoter system empty vector plasmids. (A) Plate reader growth profiles of *P. putida* TA7-EG, transformed with the three

promoter systems e.v. plasmids, in LB medium. (B) Same as (A), with growth performed in M9 minimal medium, 10 mM TA, 10 mM EG. Mean and S.D. of n=10 wells per strain are shown.

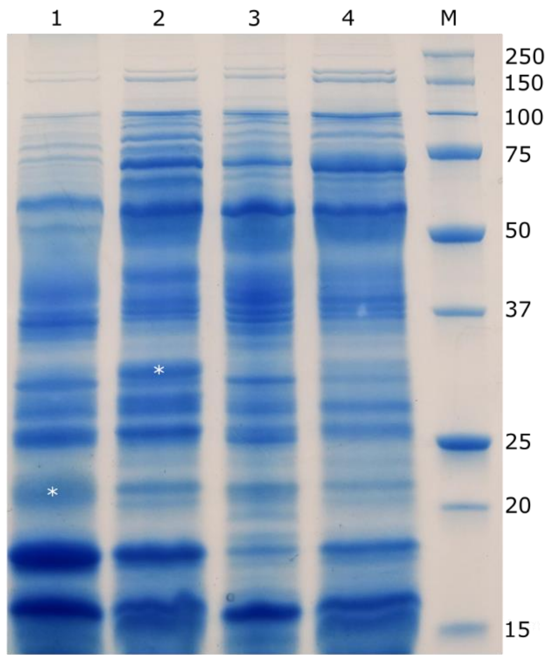

Figure S18: SDS-PAGE analysis of P_{IbpA}-bglx-HiC and P_{IbpA}-lasb-IsP^{Dura} secretion constructs. Plasmid-based or genomically integrated P_{IbpA} constructs were evaluated by SDS-PAGE and LC-MS analysis. Cultures grown in LB medium were induced by a 37°C heat-shock for 2 h and subsequently grown at 30°C for 20 h. 50 mL of culture were cleared by centrifugation and the supernatant concentrated in spin filters to ca. 500 µL volume, of which 15 µL were loaded per well. Lane 1: *P. putida* TA7-EG transformed with pLo-P_{IbpA}-bglx-HiC (expected MW 22.8 / 20.7 kDa with/without the bglx SP); Lane 2: *P. putida* TA7-EG transformed with pLo-P_{IbpA}-bglx-IsP^{Dura} (expected MW 30.4 / 27.9 kDa with/without the lasb SP); Lane 3: *P. putida* TA7-EG with P_{IbpA}-bglx-HiC genomic knock-in; Lane 4: *P. putida* TA7-EG wt; M, molecular weight marker. We observed putative bands for the plasmid-based bglx-HiC and lasb-IsP^{Dura} secretion constructs (marked by asterisks). Those bands were excised and analyzed by LC-MS, which confirmed the identity of bglx-HiC but did not unambiguously confirm the identity of lasb-IsP^{Dura}. The genomically integrated P_{IbpA}-bglx-HiC construct (lane 3) did not show a clear band in the expected size range, likely due to lower expression output compared to plasmid-based expression.

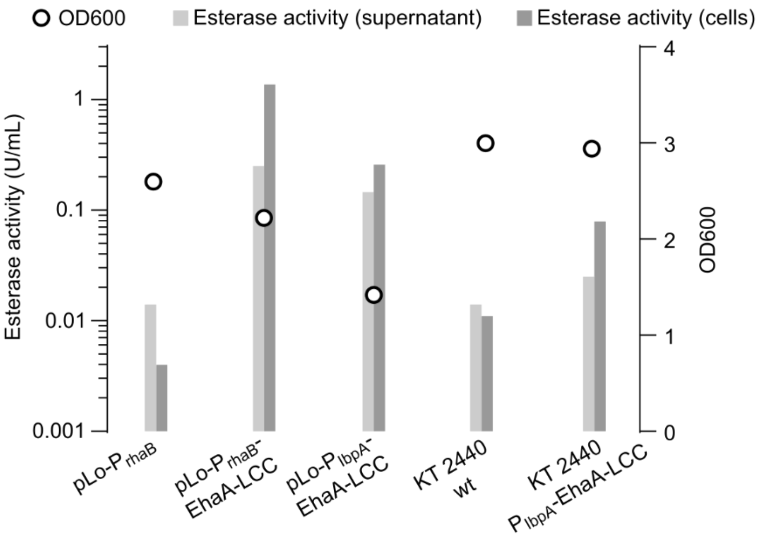

Figure S19: Comparing EhaA-LCC expression output from different promoter systems. *P. putida* TA7-EG cultures transformed with the indicated plasmids (first three entries) or *P. putida* KT2440 wt and the matching P_{IbpA}-EhaA-LCC knock-in strain were compared for EhaA-LCC expression and cell fitness. The two cultures with the P_{rhaB} plasmids were induced with 2 mM rhamnose and cultured for 3 h before analysis. The P_{IbpA} strains (both plasmid and genomic knock-in, and the KT2440 wt control) were cultured for 2 h at 37° C, followed by a 20 h outgrowth phase at 30° C before analysis. Data shown depict the mean from a single representative experiment with duplicate cultures.

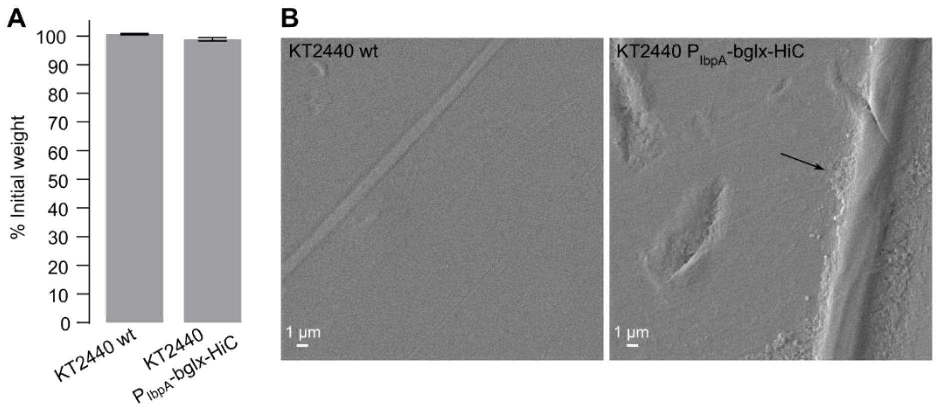

Figure S20: Testing for aPET plastic degradation with *P. putida* KT2440 P_{IbpA}-bglx-HiC. *P. putida* KT2440 wt and the corresponding P_{IbpA}-bglx-HiC knock-in strain were cultured in LB medium (15 mL in a 125 mL shake flask) for 6 days with daily medium change and supplemented with aPET film (ca. 3x3 cm). The induction regime consisted of a 2 h heat shock per day at 37° C followed by outgrowth at 25° C. (A) After 6 days, we observed slight fraying of the aPET film along the edges and a weight loss in the range of 1.5% for those samples cultured with *P. putida* P_{IbpA}-bglx-HiC. (B) SEM analysis of the film did not reveal major changes in surface structure compared to the wt control, albeit some potential pitting (arrow) of the surface especially along preexisting edges and scratches.

II Supplementary Tables

Table S1: Genomic landing sites of knock-in cassettes.

**Strain**	***tphII* cassette**	**P_{IbpA}-PET hydrolase cassette**	**Gene(s) affected**
KT2440	n.a.	n.a.	n.a.
TA	5,251,766 - 5,251,774	n.a.	PP_4627 (*cidR*)
TA7	5,251,766 - 5,251,774	n.a.	PP_4627 (*cidR*)
KT2440 P_{IbpA}-bglx-HiC	n.a.	6,055,981 - 6,055,989	PP_5309 (*oxyR*)
KT2440 P_{IbpA}-EhaA-LCC	n.a.	1,310,650 - 1,310,658	PP_1144
KT2440 P_{IbpA}-Iasb-IsP^{Dura}	n.a.	4,242,500- 4,242,508	PP_3719
TA7-BD P_{IbpA}-bglx-HiC	5,251,766 - 5,251,774	256,383 - 256,391	PP_4627, PP_0205
TA7-BD P_{IbpA}-EhaA-LCC	5,251,766 - 5,251,774	866,370 - 866,378	PP_4627, PP_0747
TA7-BD P_{IbpA}-Iasb-IsP^{Dura}	5,251,766 - 5,251,774	2,401,350 - 2,401,358	PP_4627, PP_2102
TA7-EG P_{IbpA}-bglx-HiC	5,251,766 - 5,251,774	5,323,278 - 5,323,286	PP_4627, intergenic between PP_4683 and PP_4684
TA7-EG P_{IbpA}-EhaA-LCC	5,251,766 - 5,251,774	4,505,576 - 4,505,584	PP_4627, PP_3997

Table S2: Whole-genome sequencing of engineered *P. putida* strains.

POS	TYPE	REF	ALT	FTYPE	STRAND	NT_POS	AA_POS	EFFECT	LOCUS	PRODUCT	1	2	3	4	5	6	7	8	9	10	11	12
278742	ins	T	TA	NA	NA	NA	NA	NA	NA	NA	+	+	+	+	+	+	+	+	+	+	+	+
307865	ins	G	GC	pseudo	+	NA	NA	intragenic_variant n.307865_307866insC	PP_0253	pck (pseudogene)	+	+	+	+	+	+	+	+	+	+	+	+
336124	ins	A	AT	CDS	+	211/237	71/78	frameshift_variant c.210dupT p.Thr71fs	PP_0278	hypothetical protein	+	+	+	+	+	+	+	+	+	+	+	+
353067	snp	A	G	NA	NA	NA	NA	NA	NA	NA	+	+	+	+	+	+	+	+	+	+	+	+
499203	snp	A	G	NA	NA	NA	NA	NA	NA	NA	+	+	+	+	+	+	+	+	+	+	+	+
1070246	ins	T	TGA	NA	NA	NA	NA	NA	NA	NA	+	+	+	+	+	+	+	+	+	+	+	+
1126645	ins	A	AC	NA	NA	NA	NA	NA	NA	NA	+	+	-	+	+	+	+	-	+	+	+	+
1419548	ins	G	GC	NA	NA	NA	NA	NA	NA	NA	+	+	+	+	+	+	+	+	+	+	+	+
1499480	mnp	CA	AC	NA	NA	NA	NA	NA	NA	NA	+	+	+	+	+	+	+	+	+	+	+	-
1499480	complex	CA	AC	NA	NA	NA	NA	NA	NA	NA	-	-	-	-	-	-	-	-	-	-	-	+
1499498	complex	T	CG	NA	NA	NA	NA	NA	NA	NA	+	+	+	+	+	+	+	+	+	+	+	+
1499506	ins	G	GC	NA	NA	NA	NA	NA	NA	NA	+	+	+	+	+	+	+	+	+	+	+	+
1932638	ins	G	GC	NA	NA	NA	NA	NA	NA	NA	+	+	+	+	+	+	+	+	+	+	+	+
2327922	snp	A	G	CDS	-	407/924	136/307	missense_variant c.407T>C p.Val136Ala	PP_2046	LysR family transcriptional regulator	-	-	-	-	-	-	+	+	+	+	-	-
2958523	snp	C	T	CDS	+	1283/1494	428/497	missense_variant c.1283C>T p.Ala428Val	PP_2589	aldehyde dehydrogenase family protein	+	+	+	+	+	+	+	+	+	+	+	+
3319377	snp	C	T	CDS	-	769/918	257/305	missense_variant c.769G>A p.Glu257Lys	PP_2919	LysR family transcriptional regulator	-	-	-	-	+	-	-	-	-	-	-	-
3786089	snp	G	C	CDS	-	1120/1242	374/413	missense_variant c.1120C>G p.Leu374Val	PP_3349	3-(3-hydroxy-phenyl)propionate transporter MhpT	-	-	+	-	-	-	+	+	+	+	+	+
3799247	snp	C	T	CDS	+	151/471	51/156	missense_variant c.151C>T p.Arg51Cys	PP_3359	hydroxycinnamic acid degradation regulator	-	-	+	-	-	-	+	+	+	+	+	+
3845703	ins	C	CG	NA	NA	NA	NA	NA	NA	NA	+	+	+	+	+	+	+	+	+	+	+	+
3951159	complex	TTA	CTT	NA	NA	NA	NA	NA	NA	NA	+	+	+	+	+	+	+	+	+	+	+	+
4586030	complex	CTGC	TCGCG	NA	NA	NA	NA	NA	NA	NA	+	+	-	+	+	+	+	+	+	+	+	+
4586056	ins	A	AC	NA	NA	NA	NA	NA	NA	NA	+	+	-	+	+	+	+	+	+	+	+	+
4657540	snp	G	A	CDS	+	176/1782	59/593	missense_variant c.176G>A p.Arg59His	PP_4121	NADH-quinone oxidoreductase subunit C/D	-	-	-	+	-	-	-	-	-	-	-	-
4740804	del	CT	C	NA	NA	NA	NA	NA	NA	NA	-	+	-	-	-	-	-	-	-	-	-	-
4740811	complex	TATTTTTCTG	ATTTGTCCT	NA	NA	NA	NA	NA	NA	NA	+	-	+	+	+	+	+	+	+	+	+	+
4740816	complex	TTCTG	GTCCT	NA	NA	NA	NA	NA	NA	NA	-	+	-	-	-	-	-	-	-	-	-	-
4874145	snp	C	A	CDS	-	388/756	130/251	stop_gained c.388G>T p.Glu130*	PP_4283	GntR family transcriptional regulator	-	-	-	-	-	-	-	-	-	-	+	+
4978562	del	GC	G	CDS	-	37/696	13/231	frameshift_variant c.37delG p.Ala13fs	PP_4384	flagellar L-ring protein	-	-	-	-	-	-	-	-	-	-	+	+
4980585	ins	G	GGGC	NA	NA	NA	NA	NA	NA	NA	+	+	+	+	+	+	+	+	+	+	+	+
5317300	ins	G	GT	CDS	-	347/492	116/163	frameshift_variant c.347_348insA p.Ala117fs	PP_4679	acetolactate synthase small subunit	-	-	-	-	-	-	-	-	-	-	-	+
5555608	ins	G	GGCC	NA	NA	NA	NA	NA	NA	NA	+	+	+	+	+	+	+	+	+	+	+	+
5674752	complex	CG	GCC	NA	NA	NA	NA	NA	NA	NA	+	+	+	+	+	+	+	+	+	+	+	+
5681415	ins	C	CCGGG	NA	NA	NA	NA	NA	NA	NA	+	+	+	+	+	+	+	+	+	+	+	+
5871461	ins	T	TA	CDS	-	1191/2280	397/759	frameshift_variant c.1191_1192insT p.Lys398fs	PP_5145	phosphoenolpyruvate-dependent regulator	-	-	-	+	-	-	-	-	-	-	-	-
6013885	snp	T	C	NA	NA	NA	NA	NA	NA	NA	+	+	+	+	+	+	+	+	+	+	+	+

1 *P. putida* KT2440; **2** *P. putida* TA; **3** *P. putida* TA7; **4** *P. putida* KT2440 P_{IbpA}-bglx-HiC; **5** *P. putida* KT2440 P_{IbpA}-EhaA-LCC; **6** *P. putida* KT2440 P_{IbpA}-Iasb-IsP^{Dura}; **7** *P. putida* TA7-BD; **8** *P. putida* TA7-BD P_{IbpA}-bglx-HiC; **9** *P. putida* TA7-BD P_{IbpA}-EhaA-LCC; **10** *P. putida* TA7-BD P_{IbpA}-Iasb-IsP^{Dura}; **11** *P. putida* TA7-EG P_{IbpA}-bglx-HiC; **12** *P. putida* TA7-EG P_{IbpA}-EhaA-LCC

All mutations present in the reference strain **1** and all other strains (compared against the reference genome) are greyed out; random mutations (potentially sequencing artefacts) are shown in yellow; functional mutations are shown in red. + / - signs indicate presence or absence, respectively, of a given mutation.

Table S3: Signal peptide oligonucleotide library.

Oligonucleotide sequence (5' → 3')[a]	Signal peptide protein sequence[b]	Origin (GenBank Nr.), 4-letter code[c]
catgctaaggaggtCttctaATGCAGTCGAAAACCACCCGTCGTTCGTTCGTGAAGGGTCTGGCGGCAACGGGTTTGTTGGGCGGGCTCGGTATGTGGCGTGCCCCAGTGTGGGCAGTCGCTcctgcaggcCAATCCAATC	MQSKTTRRSFVKGLAATGLLGGLGMWRAPVWAVA	*P. putida*, copper resistance protein A (AAN70945.1) **copa**
catgctaaggaggtCttctaATGGGGGAGCATGATCTGAATCGCCGTCAGTTTATTAAAACCGTCGGTGTGGCCTCGGTCGCCGCTGCCGCGATGTCGCTCCCATTTGTGAAAGCAAATGCAcctgcaggcCAATCCAATC	MGEHDLNRRQFIKTVGVASVAAAAMSLPFVKANA	*P. putida*, HA62_13390 (KEX93498.1) **spat**
catgctaaggaggtCttctaATGTGTATGGACGATTGCTGTTCCTCCAGCTCGCGCCGCGACTTTCTCAAACTGGGTGCAATGTTGACGGCTGCCGGCGCTCTGCCGCTGCTCTCGTCCCTCCAGGCACGTGCCcctgcaggcCAATCCAATC	MCMDDCCSSSSRRDFLKLGAMLTAAGALPLLSSLQARA	*P. putida*, ABC transporter substrate binder (AJA16455.1) **atsb**
catgctaaggaggtCttctaATGCCACCTACCCCAACGCGCCGCACGTTCGTCAAAGGCTTGGGTGCAGCTACCGCACTGGCAGGTCTGGGGCTCTGGCGCCCGTTGGCACAAGCCcctgcaggcCAATCCAATC	MPPTPTRRTFVKGLGAATALAGLGLWRPLAQA	*P. putida*, multicopper oxidase (AMK30412.1) **muox**
catgctaaggaggtCttctaATGTCCAATCGTGAAATTTCGCGCCGGTCCTTTTTGCAAGGTGGGTTGGTGGCTGGTGTGTCCGTGACCCTCACGCCACTGTCCTCGCAAGCACTGGCGcctgcaggcCAATCCAATC	MSNREISRRSFLQGGLVAGVSVTLTPLSSQALA	*P. putida*, Tat pathway signal sequence (SDB06516.1) **tpss**
catgctaaggaggtCttctaATGAATGACTCCGAACATTTCAACCTCCAACGGCGGCGGGTGCTGATGGGTATGGGGGCAGCCGGTGTGGCCTTGGCTGGTACGGCTTTGTCGTGTCCTGCAATGGCTcctgcaggcCAATCCAATC	MNDSEHFNLQRRRVLMGMGAAGVALAGTALSCPAMA	*P putida*, deferrochelatase / peroxidase EfeB (SDB47198.1) **efeb**
catgctaaggaggtCttctaATGCCATCGCGCCGCACCTTCTTGCAGCTCTCCCTGGCTGCGGGTCTCGGTGCTAGCCTGGGGCTCCCACTGGGTCTCGCTCGTGCAcctgcaggcCAATCCAATC	MPSRRTFLQLSLAAGLGASLGLPLGLARA	*P. putida*, agmatine deiminase (APO84336.1) **agde**
catgctaaggaggtCttctaATGGCCGATGCCATGGCGAGCAGCGTGCATCGCCGTGAAGTGATTGGGTGGATGGGTATCGGCGCATTGGCGcctgcaggcCAATCCAATC	MADAMASSVHRREVIGWMGIGALA	*P. putida*, uxpA (AAN66669.1) **uxpa**
catgctaaggaggtCttctaATGAGCCGGGACACGGGGGACAATTTGGACCGTAATCACAGCGGTAACATGCCTATGGCCAATGTCATGGACGCCTATCTCTCCCGCCGGAGCATTATGCGCGGCTCCCTCGGGGCTGCTATCGCAcctgcaggcCAATCCAATC	MSRDTGDNLDRNHSGNMPMANVMDAYLSRRSIMRGSLGAAIA	*P. putida*, uxpB (CAA56976.1) **uxpb**

catgctaaggaggtCttctaATGAAAACGCCAGCGGTCTCCCATGCTTCCCGTCGTGCATTTCAACTGACCAGCGTGACCGCGTTGATGATTAGCTTGGGGCTCATTACCGCTATGGCCcctgcaggcCAATCCAATC	MKTPAVSHASRRAFQLTSVTALMISLGLITAMA	*P. putida*, Mn(II) copper oxidase A, McoA (AAN68792.1) **mcoa**
catgctaaggaggtCttctaATGAGCAATCGGGATATTTCCCGTCGGGCGTTCCTCCAAGGTGGCCTGATTGCGGGCGTGGGGGTGACGATGGCGCCTCTGGGTAGCCAAGCCTTTGCTcctgcaggcCAATCCAATC	MSNRDISRRAFLQGGLIAGVGVTMAPLGSQAFA	*P. putida*, molybdopterin binding subunit (WP_159411419.1) **mobs**
catgctaaggaggtCttctaATGATGAAATTGAGCTTGCTCGGTCTCGCGATGGGTCTGGCGTCCCAAGCTGCACTCGCTGCTcctgcaggcCAATCCAATC	MMKLSLLGLAMGLASQAALAA	*Pseudomonas*, betaglucosidase BglX (WP_010952511.1) **bglx**
catgctaaggaggtCttctaATGACCACGAAAACGTCCATTGCAAAAGCCTTGACGCTCGCAGCAGGGTTGTCGCTGGCTTCGATGCAAGCGTTTGCGcctgcaggcCAATCCAATC	MTTKTSIAKALTLAAGLSLASMQAFA	*P. putida*, alginate biosynthesis protein AlgF (AAN66902.1) **algf**
catgctaaggaggtCttctaATGCTCCGTGCCTTGATCTTGTTGGCCTTGAGCTGTCTCCTGAGCCCCGCATTCGCGcctgcaggcCAATCCAATC	MLRALILLALSCLLSPAFA	*Pseudomonas*, murein hydrolase activator EnvC (WP_010955607.1) **envc**
catgctaaggaggtCttctaATGCTCCGTGCTACGGCTTTGGCCCTCGCATGCTTGGCTAGCTCGAGCGCAATCGCTGCCcctgcaggcCAATCCAATC	MLRATALALACLASSSAIAA	*Pseudomonas*, alpha/beta hydrolase (WP_010953184.1) **abhy**
catgctaaggaggtCttctaATGACGAATTCGTTGGCTCGTCCTAGCTTGTTGGCCCTGACCGTGTCGTTTTCGATGCTGGGGGCGGCAGCACCATCGTTTGCGGCCcctgcaggcCAATCCAATC	MTNSLARPSLLALTVSFSMLGAAAPSFAA	*Pseudomonas*, copper resistance protein B (WP_049588384.1) **copb**
catgctaaggaggtCttctaATGAATCTGTTGAAACCCTTGACGCCTAGCCTCCTCGCATTGGCCTTGGCTGCAcctgcaggcCAATCCAATC	MNLLKPLTPSLLALALAA	*Pseudomonas*, TolC family (WP_010954293.1) **tolc**
catgctaaggaggtCttctaATGTTTGCAAAAGCCGTGGCAGTGTCCCTGTTGACCCTGGCAAGCGCAAGCGTGTTCGCCGCAcctgcaggcCAATCCAATC	MFAKAVAVSLLTLASASVFAA	*Pseudomonas*, azurin (WP_003249580.1) **azur**
catgctaaggaggtCttctaATGAACATCGTCCACAAAGCATTGACGACCTCGTTGTTGGCTCTGTCGGTGTCCTCGGCTTTTGCCGCTGGTTCCCCTGGCcctgcaggcCAATCCAATC	MNIVHKALTTSLLALSVSSAFAAGSPG	*P. putida*, lipase (putative) (Q88GB2) **lipu**
catgctaaggaggtCttctaATGAAACTGAAAAACACCTTGGGCTTGGCCATTGGTTCGCTTGTAGCCGCCACTTCGATTGGCGCTATGGCAcctgcaggcCAATCCAATC	MKLKNTLGLAIGSLVAATSIGAMA	*P. putida*, OmpA family protein (WP_119713436.1) **ompa**
catgctaaggaggtCttctaATGCCCTTTCATATCCATCGTGCGACCTGTTGGGCACTGACCTGCTGCCTCTCGGGGCTCTTGGCTGCACCTCAAGCATTGGCAcctgcaggcCAATCCAATC	MPFHIHRATCWALTCCLSGLLAPQALA	*P. putida*, secretion protein (AHZ75903.1) **secp**

catgctaaggaggtCttcta<u>ATGGCGAGCACCACCGTCTCCGGCATTGGCTCGGGTGTCGATACGCAATCGATTGTCAAAGCGTTGGCC</u>cctgcaggcCAATCCAATC	MASTTVSGIGSGVDTQSIVKALA	*P. putida*, flagellar filament capping protein FliD (AAN69954.1) **flid**
catgctaaggaggtCttcta<u>ATGAATCAATCGCTCGGCGTGCTGCGTCTCACCCGTGGCGTCATTGCTCTCGCTTTGGCATCGGTGGCT</u>cctgcaggcCAATCCAATC	MNQSLGVLRLTRGVIALALASVA	*Pseudomonas*, oxidized polyvinyl alcohol hydrolase PvaB (BAA94192.1) **pvab**
catgctaaggaggtCttcta<u>ATGCAACAAAACATCGAACGCAACCAAGTGAGCATGACCACGTCCCGTTTTGTGTGGGGCGCTGTGATGGCGCTGGTCGCCCTCGGTTCCGCGTCCGCTGCT</u>cctgcaggcCAATCCAATC	MQQNIERNQVSMTTSRFVWGAVMALVALGSASAA	*Pseudomonas*, PQQ-dependent polyvinyl alcohol dehydrogenase PvaA (BAA09321.1) **pvaa**
catgctaaggaggtCttcta<u>ATGAATACCTTTTCCAAGGTCCTCACCGGCACGTTGTTGGCGATGAGCATCAGCAACGCGTTTGCC</u>cctgcaggcCAATCCAATC	MNTFSKVLTGTLLAMSISNAFA	*Pseudomonas mandelii*, EstK (AEW10549.2) **estk**
catgctaaggaggtCttcta<u>ATGAACAAGAACAAAACCTTCCTCGCGGCAGCGTTGGTGGCATTGGCAGCGTCGTTTCCTGTCCACGCTGCC</u>cctgcaggcCAATCCAATC	MNKNKTFLAAALVALAASFPVHAA	*Pseudomonas aeruginosa*, lipase lipC (AAG08198.1) **lipc**
catgctaaggaggtCttcta<u>ATGAAAAAGGTCTCGACGCTGGACCTGTTGTTTGTGGCTATCATGGGTGTCTCCCCGGCGGCCTTCGCGGCT</u>cctgcaggcCAATCCAATC	MKKVSTLDLLFVAIMGVSPAAFAA	*Pseudomonas aeruginosa*, elastase (AFM37281.1) **lasb**
catgctaaggaggtCttcta<u>ATGCACAAACGCACCTATTTGAATGCGTGCTTGGTGCTGGCGTTGGCTGCTGGTGCCTCCCAAGCCTTGGCTGCTCCTGGCGCA</u>cctgcaggcCAATCCAATC	MHKRTYLNACLVLALAAGASQALAAPGA	*Pseudomonas aeruginosa*, protease IV (ABC73074.1) **prpl**
catgctaaggaggtCttcta<u>ATGAACTTTCCCCGCGCTTCCCGCCTGATGCAGGCCGCCGTTCTCGGCGGGCTGATGGCCGTGTCGGCCGCCGCCACCGCC</u>cctgcaggcCAATCCAATC	MNFPRASRLMQAAVLGGLMAVSAAATA	*Ideonella sakaiensis*, PETase (GAP38373.1) **ispe**
catgctaaggaggtCttcta<u>ATGCAAACCACGGTCACCACCATGTTGTTGGCTTCGGTCGCTCTCGCTGCG</u>cctgcaggcCAATCCAATC	MQTTVTTMLLASVALAA	*Ideonella sakaiensis*, MHETase (GAP38911.1) **ismh**

[a] The SP coding sequence is underlined; the remaining overhangs were included for cloning purposes.

[b] The first 11 SPs are classified as Tat-dependent, the remaining 19 SPs as Sec-dependent.

[c] The provided 4-letter code is not the official gene name, but a custom naming to identify the different SPs within this manuscript.

Table S4: SP library screening data.

LCC

Clone	pNPB	OD600	SP
A4	3.28	0.471	lipu
F1	3.277	0.256	ompa
E1	3.256	0.286	ispe
H9	3.23	0.235	ispe
D3	3.181	0.327	ispe
C9	3.177	0.283	flid
B10	3.135	0.268	
D8	3.087	0.243	
F8	2.958	0.266	
E7	2.947	0.265	
G11	2.934	0.251	
H12	2.866	0.277	
F10	2.856	0.262	
C11	2.854	0.542	flid
A3	2.784	0.45	efeb
C2	2.613	0.346	efeb
G2	2.569	0.255	
F7	2.553	0.255	
D7	2.542	0.23	
B12	2.429	0.278	
F4	2.426	0.289	
F12	2.339	0.248	
A9	2.181	0.259	
D11	2.04	0.264	
G1	1.992	0.251	
A5	1.988	0.238	
G8	1.93	0.191	
C12	1.897	0.391	copa
B9	1.884	0.253	
C4	1.769	0.25	
C5	1.749	0.263	
G4	1.712	0.268	
A12	1.674	0.258	
D1	1.648	0.262	
A7	1.608	0.247	
G6	1.573	0.299	
D2	1.505	0.267	
E8	1.475	0.253	
D5	1.441	0.268	
E4	1.423	0.278	
H7	1.422	0.269	
A10	1.413	0.273	
D9	1.366	0.268	
G3	1.348	0.278	
F3	1.337	0.288	
C7	1.323	0.253	
A2	1.305	0.292	
F2	1.303	0.275	
H5	1.276	0.285	
C10	1.271	0.259	
H10	1.235	0.258	
F9	1.227	0.289	

HiC

Clone	pNPB	OD600	SP
C8	2.324	0.181	bglx
A6	2.281	0.183	bglx
H11	2.265	0.185	azur
E12	2.239	0.177	lipc
H10	2.212	0.189	bglx
C7	2.199	0.194	bglx
B12	2.182	0.273	bglx
F1	2.145	0.2	n.a.
C11	2.103	0.175	
C4	2.083	0.188	
C10	2.047	0.174	
G12	2.041	0.18	
A8	1.98	0.176	
H4	1.948	0.189	
H12	1.944	0.222	mcoa
E9	1.9	0.191	
F12	1.845	0.193	
E11	1.828	0.205	
D3	1.767	0.19	
H2	1.753	0.203	
H1	1.739	0.197	
G4	1.72	0.189	
B1	1.713	0.179	
A10	1.651	0.195	
F5	1.582	0.192	
G7	1.549	0.203	
F4	1.546	0.189	
C2	1.54	0.187	
B3	1.535	0.186	
E2	1.499	0.205	
G6	1.494	0.189	
D6	1.487	0.2	
E10	1.447	0.19	
F6	1.438	0.201	
E8	1.41	0.182	
B4	1.385	0.306	prpl
C9	1.373	0.185	
G8	1.346	0.201	
G9	1.311	0.199	
E5	1.308	0.199	
D9	1.234	0.183	
F10	1.178	0.185	
C1	1.116	0.215	
G1	1.066	0.222	
D1	0.988	0.229	
F7	0.98	0.26	
E1	0.884	0.239	
F9	0.877	0.228	
E4	0.843	0.24	
G10	0.84	0.253	
F2	0.835	0.198	
C6	0.831	0.227	

IsP

Clone	pNPB	OD600	SP
B6	2.773	0.434	pvaa
C12	2.763	0.404	lipc
B8	2.716	0.361	
B4	2.705	0.359	
C7	2.694	0.396	
C11	2.676	0.371	
A12	2.673	0.391	
E11	2.589	0.387	
B11	2.576	0.599	bglx
C1	2.571	0.432	prpl
B10	2.505	0.617	n.a.
A11	2.502	0.844	agde
A4	2.495	0.652	lasb
A5	2.49	0.324	
A6	2.478	0.892	copb
F8	2.472	0.337	
C6	2.458	0.518	lasb
H6	2.458	0.308	
G12	2.457	0.355	
B9	2.447	0.561	copb
B12	2.447	0.771	copa
D12	2.446	0.507	
B5	2.437	0.331	
A8	2.43	0.697	pvab
D5	2.427	0.374	
A10	2.423	0.355	
G2	2.419	0.267	
D8	2.401	0.843	lasb
C8	2.4	0.661	
A7	2.399	0.337	
E12	2.393	0.871	agde
A3	2.389	1.258	
A9	2.389	0.5	
C4	2.386	0.494	
D7	2.376	0.381	
G10	2.376	0.348	
E10	2.374	0.711	
C10	2.372	0.768	
C2	2.37	0.317	
D2	2.365	0.434	
E9	2.354	0.689	
D6	2.349	0.371	
C9	2.348	0.352	
F6	2.343	0.389	
B3	2.336	0.862	
B7	2.334	0.851	
D10	2.328	0.771	
F9	2.328	0.855	
D11	2.322	0.342	
F12	2.315	0.861	
E7	2.311	0.803	
E6	2.307	0.547	

B11	1.202	0.209	
H3	1.162	0.259	
B5	1.121	0.268	
B4	1.118	0.302	**uxpb**
E2	1.115	0.29	
A11	1.087	0.327	**uxpb**
G12	1.077	0.252	
F11	1.064	0.299	
E3	1.015	0.287	
B6	0.958	0.296	
B3	0.935	0.256	
H4	0.853	0.255	
E10	0.694	0.321	
H1	0.647	0.311	
H2	0.628	0.354	
H6	0.574	0.347	
B7	0.542	0.365	
B1	0.54	0.581	
G9	0.526	0.315	
H11	0.519	0.362	
D10	0.515	0.364	
C1	0.507	0.378	
E12	0.501	0.341	
G5	0.492	0.329	
A8	0.489	0.304	
B8	0.482	0.283	
F5	0.478	0.353	
A6	0.456	0.54	
C8	0.456	0.334	
G10	0.454	0.335	
G7	0.437	0.387	
C6	0.428	0.37	
D4	0.388	0.752	
E9	0.387	0.612	
A1	0.38	0.816	
D12	0.38	0.763	
F6	0.376	0.777	
D6	0.374	0.607	
C3	0.372	0.73	
E5	0.367	0.733	
B2	0.364	0.772	
E11	0.358	0.756	
E6	0.278	0.206	
H8	0.267	0.288	
E3	0.812	0.194	
B9	0.731	0.224	
D2	0.662	0.154	
G5	0.649	0.163	
H8	0.624	0.385	
B5	0.554	0.207	
A3	0.551	0.234	
A12	0.543	0.171	
H3	0.541	0.222	
H7	0.526	0.407	
E6	0.511	0.231	
A5	0.483	0.2	
D10	0.453	0.222	
G3	0.451	0.229	
H9	0.449	0.23	
B11	0.437	0.225	
G2	0.437	0.536	
D5	0.432	0.225	
C5	0.425	0.224	
B7	0.413	0.23	
F3	0.407	0.22	
F8	0.341	0.32	
H6	0.334	0.632	
D11	0.327	0.309	
A9	0.323	0.721	
A4	0.321	0.272	
A7	0.315	0.501	
D7	0.314	0.541	
B2	0.31	0.741	
E7	0.308	0.656	
B6	0.305	0.617	
D8	0.3	0.667	
H5	0.3	0.546	
B10	0.298	0.509	
A11	0.296	0.419	
C12	0.292	0.761	
C3	0.291	0.731	
B8	0.289	0.642	
A1	0.288	0.75	
D4	0.287	0.722	
A2	0.286	0.638	
F11	0.285	0.56	
G11	0.279	0.996	
D12	0.269	0.735	
E8	2.304	0.397	
C5	2.3	0.893	
F11	2.289	0.836	
D9	2.264	0.5	
G11	2.262	0.625	
H10	2.262	0.362	
B1	2.249	0.876	
F10	2.245	0.504	
E4	2.2	0.301	
A2	2.199	0.763	
F1	2.199	0.861	
F7	2.192	0.858	
D4	2.188	0.872	
F4	2.185	0.573	
F2	2.182	0.791	
F5	2.18	0.363	
G9	2.179	0.885	
D1	2.174	0.311	
G6	2.174	0.885	
E2	2.168	0.897	
A1	2.156	0.782	
C3	2.149	0.894	
E5	2.141	0.296	
H12	2.127	0.355	
D3	2.126	0.294	
G8	2.119	0.864	
G3	2.112	0.777	
G5	2.093	0.844	
E3	2.083	0.284	
G7	2.078	0.366	
G1	2.072	0.666	
B2	2.061	0.887	
E1	2.058	0.269	
G4	2.056	0.833	
H11	2.054	0.624	
H1	2.042	0.815	
F3	2.038	0.308	
H7	2.025	0.871	
H2	1.955	0.356	
H4	1.955	0.851	
H9	1.9	0.374	
H3	1.896	0.863	
H8	1.832	0.375	
H5	1.813	0.341	

pNPB esterase activity was measured 3 h post-induction; OD600 was measured 20 h post-induction. The clones marked red were selected for follow-up analysis and sequencing.

n.a.: ambiguous sequencing results, typically duplicate sequence traces (non-clonal cultures).

Table S5: Plasmids used in this work.

Plasmid name[a]	Plasmid features[b]	Reference
pBAMD1-2	Ap^R, Km^R, *oriV*(R6K), mini-Tn5 transposon	[1]
pBAMD-tphII	pBAMD1-2 derivative; P_{EM7}→*tphA1*$_{II}$, *tphA2*$_{II}$, *tphA3*$_{II}$, *tphB*$_{II}$	This work
pBAMD-P_{IbpA}-EhaA-LCC	pBAMD1-2 derivative; P_{IbpA}-EhaA-LCC knock-in cassette, Km^R	This work
pBAMD-P_{IbpA}-bglx-HiC	pBAMD1-2 derivative; P_{IbpA}-bglx-HiC knock-in cassette, Km^R	This work
pBAMD-P_{IbpA}-lasb-IsP^{Dura}	pBAMD1-2 derivative; P_{IbpA}-lasb-IsP^{Dura} knock-in cassette, Km^R	This work
pPS39	Sm/Sp^R, *oriV*(pRO1600/ColE1), rhaS-rhaR-P_{rhaB}-MCS	[6]
pSEVA328	Cm^R, *oriV*(RK2), XylS-P_m	[7]
pSEVA338	Cm^R, *oriV*(pBBR1), XylS-P_m	[7]
pHi-P_{RhaB}	pPS39 derivative; Sm/Sp^R switched to Km^R	This work
pSEVA338-P_{rhaB}	pSEVA338 derivative; XylS-P_m switched to rhaS-rhaR-P_{rhaB}-MCS	This work
pSEVA328-P_{rhaB}	pSEVA328 derivative; XylS-P_m switched to rhaS-rhaR-P_{rhaB}-MCS	This work
pMe-P_{rhaB}	pSEVA338-P_{rhaB} derivative; Cm^R switched to Km^R	This work
pLo-P_{rhaB}	pSEVA328-P_{rhaB} derivative; Cm^R switched to Km^R	This work
pLo-P_{rhaB}-LCC	pLo-P_{rhaB} derivative, LCC ORF under control of P_{rhaB}	This work
pLo-P_{rhaB}-HiC	pLo-P_{rhaB} derivative, HiC ORF under control of P_{rhaB}	This work
pLo-P_{rhaB}-IsP	pLo-P_{rhaB} derivative, IsP ORF under control of P_{rhaB}	This work
pHi-P_{rhaB}-OprF	pHi-P_{rhaB} derivative; OprF membrane display anchor	This work
pHi-P_{rhaB}-OprF-LCC	pHi-P_{rhaB}-OprF with LCC cargo	This work
pHi-P_{rhaB}-OprF-HiC	pHi-P_{rhaB}-OprF with HiC cargo	This work
pMe-P_{rhaB}-OprF	pMe-P_{rhaB} derivative; OprF membrane display anchor	This work
pMe-P_{rhaB}-OprF-LCC	pMe-P_{rhaB}-OprF with LCC cargo	This work
pMe-P_{rhaB}-OprF-HiC	pMe-P_{rhaB}-OprF with HiC cargo	This work
pLo-P_{rhaB}-OprF	pLo-P_{rhaB} derivative; OprF membrane display system	This work
pLo-P_{rhaB}-OprF-LCC	pLo-P_{rhaB}-OprF with LCC cargo	This work
pLo-P_{rhaB}-OprF-HiC	pLo-P_{rhaB}-OprF with HiC cargo	This work
pLo-P_{rhaB}-OprF-IsP	pLo-P_{rhaB}-OprF with IsP cargo	This work
pHi-P_{rhaB}-EhaA-LCC	pHi-P_{rhaB} derivative with EhaA membrane display system and LCC cargo	This work
pHi-P_{rhaB}-EhaA-HiC	pHi-P_{rhaB} derivative with EhaA membrane display system and HiC cargo	This work

pMe-P_{rhaB}-EhaA-LCC	pMe-P_{rhaB} derivative with EhaA membrane display system and LCC cargo	This work
pMe-P_{rhaB}-EhaA-HiC	pMe-P_{rhaB} derivative with EhaA membrane display system and HiC cargo	This work
pLo-P_{rhaB}-EhaA-LCC	pLo-P_{rhaB} derivative with EhaA membrane display system and LCC cargo	This work
pLo-P_{rhaB}-EhaA-HiC	pLo-P_{rhaB} derivative with EhaA membrane display system and HiC cargo	This work
pLo-P_{rhaB}-EhaA-IsP	pLo-P_{rhaB} derivative with EhaA membrane display system and IsP cargo	This work
pHi-P_{rhaB}-InaV	pHi-P_{rhaB} derivative; InaV membrane display anchor	This work
pHi-P_{rhaB}-InaV-LCC	pHi-P_{rhaB}-InaV with LCC cargo	This work
pHi-P_{rhaB}-InaV-HiC	pHi-P_{rhaB}-InaV with HiC cargo	This work
pMe-P_{rhaB}-InaV	pMe-P_{rhaB} derivative; InaV membrane display anchor	This work
pMe-P_{rhaB}-InaV-LCC	pMe-P_{rhaB}-InaV with LCC cargo	This work
pMe-P_{rhaB}-InaV-HiC	pMe-P_{rhaB}-InaV with HiC cargo	This work
pLo-P_{rhaB}-InaV	pLo-P_{rhaB} derivative; InaV membrane display anchor	This work
pLo-P_{rhaB}-InaV-LCC	pLo-P_{rhaB}-InaV with LCC cargo	This work
pLo-P_{rhaB}-InaV-HiC	pLo-P_{rhaB}-InaV with HiC cargo	This work
pLo-P_{rhaB}-SP(OprF)-LCC	pLo-P_{rhaB}-EhaA-LCC derivative; EhaA coding region and GS linker removed	This work
pLo-P_{rhaB}-SPswitch-LCC	pLo-P_{rhaB}-SP(OprF)-LCC derivative; BbsI site introduced in BCD2 translational coupler	This work
pLo-P_{rhaB}-SPswitch-HiC	pLo-P_{rhaB}-SPswitch-LCC derivative; LCC cargo replaced with HiCut	This work
pLo-P_{rhaB}-SPswitch-IsP	pLo-P_{rhaB}-SPswitch-LCC derivative; LCC cargo replaced with IsP	This work
pLo-P_{rhaB}-ispe-LCC	pLo-P_{rhaB}-SPswitch-LCC derivative with ispe SP	This work
pLo-P_{rhaB}-flid-LCC	pLo-P_{rhaB}-SPswitch-LCC derivative with flid SP	This work
pLo-P_{rhaB}-efeb-LCC	pLo-P_{rhaB}-SPswitch-LCC derivative with efeb SP	This work
pLo-P_{rhaB}-bglx-HiC	pLo-P_{rhaB}-SPswitch-HiC derivative with bglx SP	This work
pLo-P_{rhaB}-mcoa-HiC	pLo-P_{rhaB}-SPswitch-HiC derivative with mcoa SP	This work
pLo-P_{rhaB}-lasb-IsP	pLo-P_{rhaB}-SPswitch-IsP derivative with lasb SP	This work
pLo-P_{rhaB}-agde-IsP	pLo-P_{rhaB}-SPswitch-IsP derivative with agde SP	This work
pLo-P_{rhaB}-copb-IsP	pLo-P_{rhaB}-SPswitch-IsP derivative with copb SP	This work
pLo-P_L	pLo-P_{rhaB} derivative; rhaS-rhaR-P_{RhaB} switched to cI857/P_L	This work

pLo-P_L-EhaA-LCC	pLo-P_L derivative with EhaA-LCC membrane display cargo	This work
pLo-P_L-bglx-HiC	pLo-P_L derivative with bglx-HiC secretion construct cargo	This work
pLo-P_{IbpA}	pLo-P_{rhaB} derivative; rhaS-rhaR-P_{rhaB} switched to P_{IbpA}	This work
pLo-P_{IbpA}-EhaA-LCC	pLo-P_{IbpA} derivative with EhaA-LCC membrane display cargo	This work
pLo-P_{IbpA}-bglx-HiC	pLo-P_{IbpA} derivative with bglx-HiC secretion construct cargo	This work
pLo-P_{IbpA}-lasb-IsP^{Dura}	pLo-P_{IbpA} derivative with lasb-IsP^{Dura} secretion construct cargo	This work
pMe-P_{rhaB}-mhpT	pMe-P_{rhaB} derivative with mhpT^{wt} ORF	This work
pMe-P_{rhaB}-mhpT^{L374V}	pMe-P_{rhaB} derivative with mhpT^{L374V} ORF	This work

[a] Naming of expression plasmids generated in this work follows cellular copy number: Hi, high copy number, *oriV*(pRO1600/ColE1); Me, medium copy number, *oriV*(pBBR1), Lo, low copy number, *oriV*(RK2).

[b] MCS multiple cloning site; Ap ampicillin; Cm chloramphenicol; Km kanamycin; Sm/Sp streptomycin/spectinomycin.

[c] The SEVA plasmid database: http://seva-plasmids.com/

All plasmid maps and sequences are available on request.

Table S6: Bacterial strains used in this work.

Strain name	Strain features[a]	Source / Reference
E. coli NEB 5-alpha	DH5α derivative; general cloning strain	NEB
E. coli CC118 *λpir*	Cloning and maintenance of *oriV*(R6K) plasmids	SEVA
P. putida KT2440	*P. putida* reference strain, ATCC #47054	ATCC
P. putida TA	*P. putida* KT2440 with *tphII* operon knock in	This work
P. putida TA7	*P. putida* TA derivative, adapted for growth in M9-TA medium at pH ≥ 7; *mhpT*^{L374V}, PP_3359^{R51C}	This work
P. putida TA7-EG	*P. putida* TA7 derivative, adapted for growth in M9-EG medium; *gclR*^{E130*}, PP_4384^{A13fs}	This work
P. putida TA7-BD	*P. putida* TA7 derivative, adapted for growth in M9-BD medium; PP_2046^{V136A}	This work
P. putida KT2440 P_{IbpA}-EhaA-LCC	*P. putida* KT2440 with P_{IbpA}-EhaA-LCC knock-in	This work
P. putida KT2440 P_{IbpA}-bglx-HiC	*P. putida* KT2440 with P_{IbpA}-bglx-HiC knock-in	This work
P. putida KT2440 P_{IbpA}-lasb-IsP^{Dura}	*P. putida* KT2440 with P_{IbpA}-lasb-IsP^{Dura} knock-in	This work
P. putida TA7-EG P_{IbpA}-EhaA-LCC	*P. putida* TA7-EG with P_{IbpA}-EhaA-LCC knock-in	This work
P. putida TA7-EG P_{IbpA}-bglx-HiC	*P. putida* TA7-EG with P_{IbpA}-bglx-HiC knock-in	This work
P. putida TA7-BD P_{IbpA}-EhaA-LCC	*P. putida* TA7-BD with P_{IbpA}-EhaA-LCC knock-in	This work
P. putida TA7-BD P_{IbpA}-bglx-HiC	*P. putida* TA7-BD with P_{IbpA}-bglx-HiC knock-in	This work
P. putida TA7-BD P_{IbpA}-lasb-IsP^{Dura}	*P. putida* TA7-BD with P_{IbpA}-lasb-IsP^{Dura} knock-in	This work

[a] See Tables S1 and S2 for details of whole-genome sequencing and landing sites of *tphII* or P_{IbpA}-PET hydrolase knock-in cassettes.

Table S7: Oligonucleotides used in this work.

Name	Sequence (5’ → 3’)[a]
OB40	GCCACTGGATGATGGACTCCTGCATTAGAAAACCTCCTTAGCATG
OB51	GCCTAGGCCGCGGCCGCGCGAATTCTTGTTGACAATTAATCATCGGCATAGTATATCGGC
OB39	CATGCTAAGGAGGTTTTCTAATGCAGGAGTCCATCATCCAGTGGC
OB54	GTAGATCCCTCGTGTGTGCGCCTGAAGTCTTATCAGGCTTGCATCTCTTGACCCATG
OB55	TAAGACTTCAGGCGCACACACGAGGGATCTACATGATCAATGAGATTCAAATTGCAGCG
OB56	GATAGTCCCTCGTGTGTGGAGCCCAGTTACTATCACAGCGGCAAAGCCATGAGGG
OB57	TAGTAACTGGGCTCCACACACGAGGGACTATCATGACCATTGTGCATCGGCGCC
OB58	AGTGTACCCTCGTGTGTGGGTCTATCGGATCATCAGACGGGCTGTGCCCCCAG
OB59	CCGATAGACCCACACACGAGGGTACACTATGAACCACCAAATCCATATCCATGATAGCG
OB53	GGGTCCGCAATTAATTAGACAAGGGTCTCAGTGATGACCCGTTGGCCCGGTAGCAAAGAC
OB179	CGACTGAGCCTTTCGTTTTATTTGATGCCTTTAATTAAGAACGGTCG
OB192	CGGCCTAGGCATATGGAATCTCCTGAATTATCATCAAGTAC
OB191	CAGGAGATTCCATATGCCTAGGCCGCGGCCGCGCG
OB189	CTACACGAAATTCATCTGGGAGAAGTACCGC
OB178	CGACCGTTCTTAATTAAAGGCATCAAATAAAACGAAAGGCTCAGTCG
OB190	GCGGTACTTCTCCCAGATGAATTTCGTGTAG
OB182	GATGCCTTTAATTAATCAGCCAAACGTCTCTTCAG
OB183	GGGCCCCCTAGGTAATCCGGAATCGCACTTACG
OB85	GACTTCCCTAGGGGGCCCAAGTTCACTTAAAAAGGAGATCAACAATGAAAGCAATTTTCG
OB91	CATTAGAAAACCTCCTTAGCATGATTAAGATG
OB92	CATCTTAATCATGCTAAGGAGGTTTTCTAATGAAACTGAAAAACACCTTGGGCTTGGC
OB93	CCTGAAAAGCTTTCAGTGGTGGTGGTGATGATGC
OB86	GCCACCCGAACCACCCGAGCCGCCCCCGCCCCCATGGCCCTGACAATGC
OB94	GGCTCCCCTGCAGGCCAACTCGGTGCG
OB95	ATGATGCCCATGGCCCGCCCGAATCCG
OB132	TATATACCTGCAGGCCAGACCAACCC
OB133	TATATACCCATGGCCCGAACAGTTCGC
OB87	GCGGGGGCGGCTCGGGTGGTTCGGGTGGCGGTGGTTCCGGTGG
OB88	CCTGAAAAGCTTTTAGAACTGCCACTTGATGCCCACCATCGCGGAGGTG
OB89	CCTGAAAAGCTTTCAGTGGTGGTGGTGATGATGCCCATGGCCGGGTCTATCGGATGAGC
OB90	CATCTTAATCATGCTAAGGAGGTTTTCTAATGAACATCGACAAGGCACTCGTCCTG
OB121	CCAGTCACGACGCTGAGGTGCGATCGCGCGGCCGCAAGCTTTTACCC
OB122	GCGACTTTCGCACGAATAACCGGCATTGTCAGGGCCATGGGTAAAAGCTTGCGGCCGCGC

OB134	CATCTTAATCATGCTAAGGAGGTCTTCTAATGAAACTGAAAAACACCTTGG
OB135	CCAAGGTGTTTTTCAGTTTCATTAGAAGACCTCCTTAGCATGATTAAGATG
OB136	CATGCTAAGGAGGTCTTC
OB137	GATTGGATTGGCCTGCAGG
OB139	ACGCTGAGGTGCGATCGCGCGGCCGC
OB211	ATCATGCTAAGGAGGTCTTCTAATGCAATCCAATCCATACCAGCGGGG
OB210	ATCATGCTAAGGAGGTCTTCTAATGCAACTCGGTGCGATTGAAAATGGTC
OB209	ATCATGCTAAGGAGGTCTTCTAATGCAGACCAACCCATACGCACGTG
OB201	CAGAGTACTTGATGATAATTCAGGAGATTCCATATGAAACTGAAAAACACCTTGGGCTTG
OB197	GATTCCATATGATGAAATTGAGCTTGCTCGGTCTC
OB195	GATTCCATATGAAAAAGGTCTCGACGCTGGACCTG
OB220	CGGCCGCAAGCTTTTACGAACAGTTCGCGGTGCG
OB221	GATGCCTTTAATTAAGAACGGTCGTTCTCAAGGCTCTTG
OB143	CCTTTCGGCGGGCTTTGGCGGCCGCAAGCTTTTACCCATGGCCCGCCCGAATCC
OB205	CGAATTCGAGCTCGAACGGTCGTTCTCAAGGCTCTTG
OB119	CCTTTCGGCGGGCTTTGGCGGCCGCAAGCTTTTAGAACTGCCACTTGATGCCCACC
OB214	CGCCTAGGCCGCGGCCGCGCGAATTCGAGCTCGAACGGTCGTTCTCAAGGCTCTTG
OB109	CGATCGCCCTAGGACGAGGGATCTACATGCATCATTCATGCGCGTCATCGG
OB110	CGGCCGCAAGCTTCTATGCAGGGTCCTGGGCTGCAG
OB96	GGCACGCGTCGACTAGTACNNNNNNNNNNACGCC
OB105	GTAAATACGCTGCTGCTCTTGGTC
OB97	GGCACGCGTCGACTAGTAC
OB104	CACTGGATGATGGACTCCTGC
OB102	ACCCATTTCGGGAATTTGCATGAC
OB103	CAGGACTTCCATGCCGACGTC

[a] Oligos are not ordered by name, but chronologically as discussed in the *Materials and Methods* section.

III Synthetic DNA sequences

Sequence S1: P_{EM7}-BCD2 promoter-bicistronic translational coupler fragment.

CTGTCTCTTATACACATCTTTGTGTCTCAGGCCGTTGTTGACAATTAATCATCGGCATAGTA
TATCGGCATAGTATAATACGACAAGGTGAGGAACTAAACCCCTAGGGCCCAAGTTCACTTA
AAAAGGAGATCAACAATGAAAGCAATTTTCGTACTGAAACATCTTAATCATGCTAAGGAGGT
TTTCTAATG

Sequence S2: *Comamonas sp*. E6, *tphA2$_{II}$*, Genbank AB238679.1 and BAE47085.1, oxygenase large subunit of terephthalate 1,2-dioxygenase, codon-optimized for *P. putida.*

ATGCAGGAGTCCATCATCCAGTGGCATGGGGCGACCAATACCCGGGTGCCGTTCGGCATT
TACACCGACACGGCGAATGCTGACCAAGAGCAGCAGCGTATTTACCGTGGTGAAGTCTGG
AACTACCTCTGCCTGGAAAGCGAAATCCCTGGTGCGGGGGACTTCCGTACGACCTTCGCG
GGCGAAACGCCCATTGTGGTGGTCCGCGACGCTGACCAGGAAATTTATGCATTCGAAAAT
CGGTGCGCTCATCGTGGGGCGCTGATCGCTCTCGAAAAAAGCGGCCGTACGGACTCGTTT
CAATGCGTCTACCATGCCTGGAGCTATAATCGTCAGGGGGATTTGACCGGGGTCGCTTTT
GAAAAGGGGGTGAAGGGGCAAGGTGGTATGCCGGCGTCCTTTTGTAAAGAGGAACATGGT
CCTCGTAAGTTGCGCGTGGCCGTCTTTTGTGGTTTGGTCTTTGGCTCCTTCTCGGAGGATG
TGCCATCGATTGAGGACTATCTGGGGCCAGAAATCTGCGAGCGGATCGAACGTGTCCTCC
ATAAGCCAGTCGAAGTCATTGGCCGTTTCACGCAAAAGCTCCCGAACAACTGGAAGCTGTA
TTTCGAAAACGTGAAGGATAGCTATCATGCCTCCCTCTTGCACATGTTTTTTACGACGTTCG
AGTTGAATCGGCTCTCCCAGAAGGGGGGCGTGATCGTCGATGAATCGGGTGGTCACCAC
GTCTCGTATAGCATGATCGACCGGGGGGCGAAGGATGACTCGTACAAGGATCAGGCCATC
CGTTCCGACAATGAACGTTATCGTCTGAAAGACCCGTCGCTCCTCGAGGGCTTTGAGGAAT
TTGAGGACGGTGTCACCCTCCAGATCTTGTCGGTGTTCCCGGGTTTTGTGCTCCAGCAGAT
CCAAAATAGCATCGCCGTGCGTCAATTGCTGCCTAAGAGCATCAGCTCGTCGGAATTGAAT
TGGACCTATCTGGGGTATGCGGACGATTCGGCGGAACAACGGAAGGTGCGCTTGAAACAG
GCCAATCTCATCGGTCCCGCCGGTTTCATCTCCATGGAAGATGGGGCGGTGGGGGGCTTC
GTCCAACGGGGGATTGCCGGTGCAGCGAATTTGGACGCCGTGATCGAGATGGGTGGGGA
CCATGAAGGGTCCTCCGAGGGCCGCGCCACGGAGACCAGCGTGCGTGGGTTTTGGAAGG
CGTACCGTAAGCACATGGGTCAAGAGATGCAAGCCTGA

Sequence S3: *Comamonas sp*. E6, *tphA3*$_{II}$, Genbank AB238679.1 and BAE47086.1, oxygenase small subunit of terephthalate 1,2-dioxygenase, codon-optimized for *P. putida.*

ATGATCAATGAGATTCAAATTGCAGCGTTTAACGCTGCGTATGCCAAAACCATCGACTCCG
ATGCTATGGAACAGTGGCCGACCTTCTTCACGAAAGATTGCCATTATTGCGTGACCAACGT
CGATAACCACGATGAGGGTCTGGCCGCTGGCATTGTCTGGGCTGACTCCCAAGATATGTT
GACCGACCGCATTTCCGCTTTGCGCGAGGCCAATATCTACGAACGGCACCGCTACCGTCA
TATCCTGGGTCTGCCGTCCATTCAGTCGGGGGACGCAACGCAAGCGTCCGCCTCCACGC
CGTTCATGGTCCTCCGCATTATGCACACGGGCGAAACGGAGGTGTTCGCGTCGGGCGAAT
ACCTCGACAAATTCACGACCATCGATGGTAAACTGCGCCTCCAGGAGCGGATCGCAGTCT
GCGACTCCACCGTGACCGATACCCTCATGGCTTTGCCGCTGTGA

Sequence S4: *Comamonas sp*. E6, *tphB*$_{II}$, Genbank AB238679.1 and BAE47087.1, 1,2-dihydroxy-3,5-cyclohexadiene-1,4-dicarboxylate dehydrogenase, codon-optimized for *P. putida.*

ATGACCATTGTGCATCGGCGCCTCGCACTCGCAATTGGTGATCCGCACGGTATTGGTCCA
GAGATTGCGCTCAAAGCGCTGCAACAACTGAGCGTCACGGAACGCTCGCTGATTAAAGTG
TATGGCCCATGGTCCGCGTTGGAACAAGCCGCTCGTGTCTGCGAAATGGAACCTTTGTTG
CAAGACATTGTCCATGAAGAGGCAGGGACCCTGACGCAGCCCGTGCAGTGGGGCGAAAT
CACGCCACAGGCTGGCCTCTCCACGGTGCAATCCGCGACGGCCGCGATCCGCGCTTGTG
AGAACGGCGAAGTGGATGCCGTGATTGCCTGCCCGCATCACGAGACGGCCATCCACCGT
GCAGGCATCGCGTTCTCCGGGTACCCGTCCCTGTTGGCGAACGTCCTCGGTATGAATGAG
GACCAAGTGTTTCTGATGCTGGTGGGCGCCGGTCTCCGCATTGTCCACGTCACGCTCCAC
GAGAGCGTGCGTAGCGCATTGGAGCGTTTGTCGCCGCAGCTGGTGGTCAATGCTGCGCA
AGCAGCGGTCCAAACGTGCACCCTCTTGGGTGTCCCTAAGCCCAAAGTCGCAGTCTTCGG
TATTAATCCTCATGCAAGCGAGGGCCAGCTGTTCGGCCTGGAGGATTCGCAGATCACCGT
CCCGGCAGTCGAAACGTTGCGCAAACGCGGCTTGGCGGTGGATGGTCCAATGGGCGCCG
ACATGGTGCTCGCGCAGCGGAAACATGATCTGTATGTGGCAATGCTGCACGACCAAGGCC
ACATCCCCATTAAATTGCTCGCACCCAATGGCGCTAGCGCCCTCTCCATTGGGGGTCGCG
TCGTCCTGTCGTCCGTCGGCCATGGGTCGGCGATGGATATCGCTGGTCGTGGTGTCGCTG
ATGCCACCGCGCTGCTGCGTACCATTGCATTGCTGGGGGCACAGCCCGTCTGA

Sequence S5: *Comamonas sp*. E6, *$tphA1_{II}$*, Genbank AB238679.1 and BAE47088.1, reductase component of terephthalate 1,2-dioxygenase, codon-optimized for *P. putida.*

ATGAACCACCAAATCCATATCCATGATAGCGACATCGCGTTCCCGTGCGCTCCTGGTCAGA
GCGTCCTCGATGCAGCACTCCAGGCAGGGATTGAATTGCCGTACTCCTGTCGTAAAGGCT
CGTGCGGGAACTGCGCCTCGACCTTGTTGGACGGTAATATTGCATCCTTTAATGGCATGG
CAGTCCGTAATGAGTTGTGTGCCTCCGAGCAAGTGCTCCTGTGTGGGTGTACCGCCGCGA
GCGATATCCGGATTCACCCATCCTCGTTCCGCCGGCTGGACCCAGAAGCTCGCAAACGGT
TCACCGCGAAAGTCTATTCGAATACGCTCGCCGCGCCCGATGTCTCCTTGTTGCGGCTGC
GGCTGCCAGTGGGTAAACGCGCAAAATTTGAAGCAGGTCAGTACCTCTTGATCCACCTCG
ATGATGGCGAATCGCGGTCGTACTCCATGGCGAATCCCCCTCATGAGTCGGATGGTATCA
CGCTGCACGTGCGGCACGTCCCGGGTGGGCGTTTCAGCACCATCGTGCAACAACTGAAAA
GCGGGGATACGTTGGACATCGAATTGCCGTTCGGTTCCATCGCCTTGAAGCCGGATGATG
CTCGTCCTCTCATCTGTGTGGCCGGTGGTACCGGGTTTGCACCTATTAAGTCGGTCCTCGA
CGACTTGGCGAAACGGAAAGTGCAACGGGATATTACGCTGATTTGGGGCGCTCGTAATCC
TAGCGGCCTCTACTTGCCAAGCGCGATCGACAAATGGCGCAAAGTCTGGCCACAGTTCCG
TTACATCGCAGCTATCACGGATCTCGGGGATATGCCGGCTGATGCTCACGCTGGCCGCGT
GGACGATGCATTGCGCACCCATTTCGGGAATTTGCATGACCACGTGGTGCACTGCTGTGG
TTCCCCCGCGTTGGTCCAATCGGTGCGGACGGCCGCGAGCGACATGGGGTTGCTGGCTC
AGGACTTCCATGCCGACGTCTTTGCTACCGGGCCAACGGGTCATCACTGA

Sequence S6: P_{IbpA} oligo (ssDNA) fragment, including parts of the T1 terminator and PacI site at the 5' end for cloning purposes (the actual P_{IbpA} fragment is underlined).

gatgcctttaatTAAGAACGGTCGTTCTCAAGGCTCTTGAAATACTTCCGACGATCCCTATCTAGT
TCGTGCGGGATGCCGAAGACGGGTTCCGCATCAGAGTACTTGaTGATAATTCAGGAGATTC

Sequence S7: cI857/P_L fragment, adapted for the SEVA plasmid system (based on Aparicio *et al*., 2019).[8]

TCAGCCAAACGTCTCTTCAGGCCACTGACTAGCGATAACTTTCCCCACAACGGAACAACTC
TCATTGCATGGGATCATTGGGTACTGTGGGTTTAGTGGTTGTAAAAACACCTGACCGCTAT
CCCTGATCAGTTTCTTGAAGGTAAACTCATCACCCCCAAGTCTGGCTATGCAGAAATCACC
TGGCTCAACAGCCTGCTCAGGGTCAACGAGAATTAACATTCCGTCAGGAAAGCTCGGCTT

GGAGCCTGTTGGTGCGGTCATGGAATTACCTTCAACCTCAAGCCAGAATGCAGAATCACTG
GCTTTTTTGGTTGTGCTTACCCATCTCTCCGCATCACCTTTGGTAAAGGTTCTGAGCTTAGG
TGAGAACATCCCTGCCTGAACATGAGAAAAAACAGGGTACTCATACTCACTTCTAAGTGAC
GGCTGCATACTAACCGCTTCATACATCTCGTAGATTTCTCTGGCGATTGAAGGGCTAAATT
CTTCAACGCTAACTTTGAGAATTTTTGTAAGCAATGCGGCGTTATAAGCATTTAATGCATTG
ATGCCATTAAATAAAGCACCAACGCCTGACTGCCCCATCCCCATCTTGTCTGCGACAGATT
CCTGGGATAAGCCAAGTTCATTTTTCTTTTTTTCATAAATTGCTTTAAGGCGGCGTGCGTCC
TCAAGCTGCTCTTGTGTTAATGGTTTCTTTTTTGTGCTCATGCTAGCTTTTTCCTCCTTATAA
AGTTAATCAACACCCCTTGTATTACTGTTTATGTAAGCAGACAGTTTTATTGTTCATGATGAT
ATATTTTTATCTTGTGCAATGTAACATCAGAGATTTTGAGACACAAGACGTGGCAAAAAACA
TTATCCAGAACGGGAGTGCGCCTTGAGCGACACGAATTATGCAGTGATTTACGACCTGCAC
AGCCATACCACAGCTTCCGATGGCTGCCTGACGCCAGAAGCATTGGTGCACCGTGCAGTC
GATGATAAGCTGTCAAACAGCGGATAACAATTTCACACAGGATAACCCGGCCTCAGCGCC
GGGTTTTCTTTGCCTCACGATCGCCCCCAAAACACATAACCAATTGTATTTATTGAAAAATA
AATAGATACAACTCACTAAACATAGCAATTCAGATCTCTCACCTACCAAACAATGCCCCCCT
GCAAAAAATAAATTCATATAAAAAACATACAGATAACCATCTGCGGTGATAAATTATCTCTG
GCGGTGTTGACATAAATACCACTGGCGGTGATACTGAGCACATCAGCAGGACGCACTGAC
CACCATGAAGGTGACGCTCTTAAAAATTAAGCCCTGAAGAAGGGCAGCATTCAAAGCAGAA
GGCTTTGGGGTGTGTGATACGAAACGAAGCATTGGCCGTAAGTGCGATTCCGGATTA

Sequence S8: BCD2-Leaf compost cutinase (LCC) fragment (LCC: residues 35-293, Uniprot G9BY57, GenBank AEV21261.1), including the N-terminal BCD2 bicistronic linker, followed by residues 1-37 of *P. putida* OprF (as described by Tozakidis *et al*., 2019)[9], a 6xHis tag, and a PstI restriction site, codon-optimized for *P. putida.*

cacttaaaaaggagatcaacaatgaaagcaattttcgtactgaaacatcttaatcatgctaaggaggttttctaATGAAACTGAA
AAACACCTTGGGCTTGGCCATTGGTTCGCTTGTAGCCGCCACTTCGATTGGCGCTATGGC
ACAAGGTCAAGGCGCCGTCGAGACTGAAATCTTCTACAAGCATCATCACCACCACCACcctg
caggcCAATCCAATCCATACCAGCGGGGTCCAAATCCTACGCGTTCCGCGCTGACGGCAGA
CGGGCCGTTCTCCGTCGCTACCTATACCGTCTCCCGTCTCTCCGTGTCCGGTTTCGGCGG
CGGTGTCATCTACTATCCCACGGGTACCAGCCTGACCTTCGGGGGGATTGCCATGTCGCC
AGGCTATACGGCAGATGCCAGCTCCTTGGCCTGGCTCGGCCGTCGTCTCGCTTCCCACGG
TTTCGTCGTCCTCGTCATCAACACGAACTCGCGTTTTGATTATCCTGATTCGCGGGCGTCG
CAGCTGAGCGCTGCGCTGAACTATTTGCGGACGAGCTCCCCGTCCGCAGTGCGTGCACG

TCTCGACGCAAATCGTTTGGCCGTCGCAGGGCACAGCATGGGTGGCGGGGGTACGTTGC
GGATCGCAGAGCAGAATCCCTCGCTCAAAGCGGCAGTCCCGCTCACGCCGTGGCACACC
GACAAGACCTTCAATACGTCCGTCCCAGTCCTCATCGTCGGCGCTGAGGCCGATACGGTG
GCCCCTGTGTCCCAGCACGCAATTCCATTTTACCAGAACCTGCCCTCCACCACGCCTAAAG
TCTATGTCGAACTGGACAACGCTTCGCATTTTGCCCCTAATAGCAACAATGCAGCAATTTCC
GTGTACACGATTTCGTGGATGAAACTGTGGGTCGACAACGATACGCGGTATCGGCAGTTC
CTCTGCAATGTCAATGACCCCGCTCTGAGCGACTTTCGCACGAATAACCGGCATTGTCAGg
gccatgggGG

Sequence S9: *Pseudomonas putida* KT2440 OprF (Uniprot Q88L46, GenBank AAN67703.1), residues 1-206, including a C-terminal $G_4SG_2S(G_4S)_3$ linker, PstI and NcoI restriction sites, 6xHis tag, and HindIII restriction site.

ATGAAACTGAAAAACACCTTGGGCTTGGCCATTGGTTCGCTTGTAGCCGCCACTTCGATTG
GCGCTATGGCACAAGGTCAAGGCGCCGTCGAGACTGAAATCTTCTACAAGAAAGAgTTCTT
CGACAGCCAGCGCGACTTCAAGAACGACGGCAACCTGTTCGGCGGCTCGATCGGTTACTT
CCTGACCGACGACGTTGAGCTGCGTCTGGGCTACGACGAAGTGCACAACGCTCGTGGCG
AAGACGGCAAGAACATCAAGGGCTCGAACACTGCCCTGGACGCCGTTTACCACTTCAACA
ACCCGTACGACGCTATCCGTCCATACGTTTCCGCTGGTTTCTCGCACCAGTCGCTGGGtCA
GACCGGCCGTGGCGGTCGTGACCACTCCACCTTCGCCAACGTTGGCGCTGGCGCCAAGT
GGTACATCACCGACATGTTCTATGCCCGTGCCGGCGTAGAAGCTCAGTACAACATCGACC
AGGGCGACACCGAGTGGGCCCCGAGCGTTGGTGTTGGCCTGAACTTCGGCGGTAGCCCG
AAGCAAGCTGAAGCTGCTCCTGCTCCAGTTGCTGAAGTCTGCTCCGACTCCGACAACGAC
GGCGTtTGCGAtAAtGTCGGcGGtGGtGGCagcGGTGGcTCcGGcGGcGGTGGTagtGGTGGcG
GtGGTTCCGGCGGtGGCGGCagccctgcaggcTCATCCGATAGACCCggccatgggCATCATCACC
ACCACCACtgaaagctt

Sequence S10: *Humicola insolens* cutinase (HiC) (Uniprot A0A075B5G4, GenBank ASK40094.1), including a N-terminal PstI and a C-terminal NcoI restriction site, codon-optimized for *P. putida.*

cctgcaggcCAACTCGGTGCGATTGAAAATGGTCTCGAAAGCGGCTCGGCGAATGCGTGCCC
AGACGCCATTCTGATTTTCGCTCGTGGTTCGACCGAACCCGGTAACATGGGGATCACGGT

GGGCCCAGCTTTGGCCAACGGTTTGGAATCGCACATCCGTAACATTTGGATCCAAGGTGT
GGGTGGCCCCTACGACGCGGCACTCGCTACGAATTTTCTCCCACGTGGTACCAGCCAGGC
GAATATTGACGAAGGTAAACGTCTGTTTGCATTGGCGAATCAGAAGTGTCCCAATACCCCT
GTCGTCGCGGGGGGCTATTCCCAAGGTGCGGCGCTCATCGCTGCGGCTGTGTCCGAATT
GTCCGGCGCCGTGAAGGAGCAAGTGAAAGGTGTGGCTCTCTTCGGGTATACCCAGAATCT
CCAAAATCGGGGTGGCATCCCTAATTACCCGCGTGAACGGACGAAGGTGTTTTGCAACGT
CGGGGATGCCGTCTGCACCGGCACCCTGATTATTACCCCTGCTCATCTGTCGTATACCATT
GAGGCTCGCGGTGAGGCAGCCCGGTTCTTGCGCGATCGGATTCGGGCGggccatggg

Sequence S11: *Ideonella sakaiensis* PETase, residues 28-290 (Uniprot A0A0K8P6T7, GenBank BBYR01000074.1), codon-optimized for *P. putida.*

CAGACCAACCCATACGCACGTGGTCCCAATCCTACCGCTGCCAGCCTCGAAGCCAGCGCG
GGCCCATTTACGGTGCGTTCGTTTACGGTGTCCCGGCCTAGCGGTTATGGGGCGGGCAC
CGTCTACTATCCAACGAATGCTGGCGGGACGGTCGGCGCcATCGCAATTGTCCCTGGTTA
CACGGCTCGCCAGTCCAGCATCAAATGGTGGGGGCCGCGGCTCGCATCCCACGGTTTTG
TGGTGATTACGATTGACACGAACTCGACCCTCGACCAGCCTAGCTCGCGGTCGTCGCAAC
AAATGGCCGCGCTGCGCCAAGTGGCATCCCTCAATGGCACCAGCTCcAGCCCGATCTATG
GCAAAGTCGATACGGCACGTATGGGGGTGATGGGTTGGTCGATGGGCGGTGGCGGTTCG
TTGATCTCCGCAGCGAACAATCCGTCCTTGAAGGCAGCTGCACCACAAGCGCCCTGGGAT
TCGTCGACGAACTTTTCGAGCGTCACGGTGCCGACCTTGATTTTCGCCTGTGAGAACGATT
CCATTGCACCCGTGAACTCGTCCGCCCTGCCAATCTACGATTCCATGTCGCGTAATGCAAA
GCAGTTCTTGGAGATTAATGGGGGTTCGCACAGCTGCGCCAACTCCGGGAACAGCAACCA
GGCCTTGATTGGTAAGAAAGGGGTCGCTTGGATGAAGCGTTTCATGGACAATGACACCCG
TTATAGCACGTTCGCGTGTGAGAATCCGAACTCGACCCGcGTgAGCGATTTTCGCACCGCG
AACTGTTCG

Sequence S12: *Escherichia coli* EhaA, residues 840-1323 (Uniprot A0A1Y2Y633, GenBank WP_023307657.1), including parts of an N-terminal $G_4SG_2S(G_4S)_3$ linker. Note: the last four 3'-terminal bases of EhaA were omitted to keep the fragment at 1500 bases to reduce synthesis cost and were re-introduced with primer OB88 during PCR amplification of this fragment; codon-optimized for *P. putida*.

CGGGTGGCGGTGGTTCCGGTGGcGGGGGTTCCGGCGGGGGCGGCTCCCTCACCAATAAT
GGTACgCTGATGACCGGCATGTCCGGTCAGCAAGCCGGTAACGTCTTGGTCGTGAAGGGC
AACTACCACGGTAACAATGGGCAACTCGTGATGAATACCGTGTTGAATGGTGATGATAGCG
TCACCGATAAACTCGTCGTGGAAGGCGACACCTCCGGCACCACGGCGGTCACGGTCAATA
ACGCTGGCGGTACgGGCGCTAAGACGCTGAACGGGATTGAACTGATTCATGTGGATGGGA
AGTCCGAAGGTGAATTTGTCCAGGCTGGTCGGATTGTGGCAGGGGCTTACGACTATACGC
TCGCTCGCGGCCAGGGCGCGAACTCGGGGAACTGGTATCTGACCTCGGGTTCGGATTCG
CCAGAGCTGCAACCCGAGCCAGATCCGATGCCGAATCCAGAGCCTAACCCAAATCCGGAA
CCAAACCCTAATCCCACCCCGACGCCGGGTCCAGATTTGAACGTCGATAATGATCTCCGC
CCAGAGGCTGGCTCCTACATTGCGAACCTCGCTGCTGCGAATACGATGTTCACCACCCGC
CTCCACGAACGGCTCGGCAACACGTACTACACGGACATGGTCACGGGCGAACAAAAGCAA
ACCACGATGTGGATGCGGCACGAAGGTGGGCACAACAAATGGCGTGACGGGAGCGGTCA
GCTGAAGACCCAAAGCAATCGCTATGTGTTGCAACTCGGGGGCGATGTGGCGCAGTGGA
GCCAAAATGGGTCGGACCGTTGGCACGTCGGGGTCATGGCCGGGTATGGGAATAGCGAC
TCCAAAACGATCAGCAGCCGCACCGGCTATCGCGCTAAGGCCTCGGTCAACGGTTACTCC
ACGGGCTTGTATGCGACGTGGTATGCCGACGATGAGTCGCGCAACGGCGCTTACCTCGAC
AGCTGGGCGCAATACTCCTGGTTCGACAACACGGTCAAGGGTGATGATCTGCAAAGCGAG
TCCTACAAGTCCAAAGGGTTCACCGCAAGtTTGGAAGCAGGGTATAAGCATAAATTGGCGG
AgTTCAACGGcTCCCAGGGcACCCGGAATGAGTGGTACGTCCAGCCTCAAGCGCAGGTGA
CGTGGATGGGCGTCAAAGCCGACAAACACCGGGAGAGCAATGGTACGCTCGTCCACAGC
AACGGCGACGGCAATGTCCAAACGCGTCTCGGCGTGAAGACCTGGTTGAAGAGCCATCAC
AAGATGGACGACGGGAAATCGCGTGAGTTTCAACCCTTCGTGGAGGTGAATTGGCTGCAC
AACAGCAAAGACTTTTCGACGTCgATGGACGGTGTCTCCGTGACGCAGGACGGGGCCCGC
AATATCGCTGAAATTAAAACGGGTGTGGAAGGTCAGCTCAATGCTAACCTCAACGTGTGGG
GCAATGTCGGGGTGCAGGTCGCGGACCGGGGCTACAACGACACCTCCGCgATGGTGGGC
ATCA

Sequence S13: *Pseudomonas syringae* InaV (Uniprot O33479, GenBank AJ001086), encompassing the N-terminal 609 bases followed by the C-terminal 291 bases; the fragment also encodes a C-terminal $G_4SGGS(G_4S)_3$ linker and PstI / NcoI restriction sites, codon-optimized for *P. putida.*

ATGAACATCGACAAGGCACTCGTCCTGCGTACCTGCGCGAACAACATGGCGGACCACTGC
GGCCTGATTTGGCCCGCCAGCGGTACGGTGGAGTCCAAGTACTGGCAATCCACGCGGCG

CCATGAAAACGGGCTCGTGGGCTTGCTGTGGGGTGCAGGGACCTCGGCGTTTTTGAGCG
TGCACGCGGATGCCCGCTGGAAAGTGTGTGAAGTCGCGGTGGCCGACATCATTGGGCTG
GAAGAACCAGGCATGGTCAAATTTCCACGGGCGGAAGTCGTCCATGTCGGTGACCGCATT
TCGGCGAGCCATTTCATTAGCGCGCGGCAGGCCGATCCTGCCAGCACGCCAACCCCTAC
CCCAACGCCGATGGCGACCCCCACCCCAGCCGCAGCCAATATTGCTTTGCCAGTCGTCGA
GCAGCCGTCCCACGAAGTCTTTGACGTCGCCCTGGTGAGCGCAGCTGCTCCGTCCGTCAA
CACCCTCCCTGTCACGACGCCTCAAAACTTGCAAACCGCCACGTACGGTTCGACGTTGTC
GGGCGACAATAATTCCCGCCTGATTGCCGGCTACGGGTCCAATGAGACCGCGGGCAATCA
TTCCGATCTGATCGGCGGCCACGACTGTACCCTGATGGCGGGCGACCAGAGCCGCCTCA
CGGCTGGCAAAAACAGCATCCTGACGGCAGGGGCGCGTAGCAAACTCATCGGCTCGGAG
GGTTCCACGTTGTCCGCTGGGGAGGATTCGACGTTGATCTTCCGCCTCTGGGACGGGAAA
CGCTACCGTCAACTCGTGGCTCGTACGGGCGAGAACGGTGTGGAGGCAGACATTCCGTA
CTACGTCAACGAGGATGACGACATCGTCGATAAGCCGGACGAAGATGACGACTGGATCGA
AGTGGAGGGtGGtGGCGGCTCcGGTGGTTCGGGcGGcGGTGGTTCCGGTGGcGGtGGTTCC
GGCGGtGGCGGCTCCcctgcaggcTCATCCGATAGACCCggccatg

Sequence S14: P_{IbpA}-IsP^{Dura} fragment, including the N-terminal P_{IbpA} promoter-RNAT unit, followed by the ORF of DuraPETase as described by Cui *et al.*, 2021;[10] codon-optimized for *P. putida*.

GAACGGTCGTTCTCAAGGCTCTTGAAATACTTCCGACGATCCCTATCTAGTTCGTGCGGGA
TGCCGAAGACGGGTTCCGCATCAGAGTACTTGaTGATAATTCAGGAGATTCcatATGAAAAA
GGTCTCGACGCTGGACCTGTTGTTTGTGGCTATCATGGGTGTCTCCCCGGCGGCCTTCGC
GGCTcctgcaggcCAGACCAACCCATACGCACGTGGTCCCAATCCTACCGCTGCCAGCCTCG
AAGCCAGCGCGGGCCCATTTACGGTGCGTTCGTTTACGGTGTCCCGGCCTAGCGGTTATG
GGGCGGGCACCGTCTACTATCCAACGAATGCTGGCGGGACGGTCGGCGCcATCGCAATT
GTCCCTGGTTACACGGCTCGCCAGTCCAGCATCAAATGGTGGGGGCCGCGGCTCGCATC
CCACGGTTTTGTGGTGATTACGATTGACACGAACTCGACCttcGACtacCCTAGCTCGCGGTC
GTCGCAACAAATGGCCGCGCTGCGCCAAGTGGCATCCCTCAATGGCgacAGCTCcAGCCC
GATCTATGGCAAAGTCGATACGGCACGTATGGGGGTGATGGGTcacTCGATGGGCGGTGG
CgccTCGTTGcgcTCCGCAGCGAACAATCCGTCCTTGAAGGCAGCTatcCCACAAGCGCCCTG
GGATTCGcagACGAACTTTTCGAGCGTCACGGTGCCGACCTTGATTTTCGCCTGTGAGAAC
GATTCCATTGCACCCGTGAACTCGcacGCCCTGCCAATCTACGATTCCATGTCGCGTAATGC
AAAGCAGTTCTTGGAGATTAATGGGGGTTCGCACAGCTGCGCCAACTCCGGGAACAGCAA

CCAGGCCTTGATTGGTAAGAAAGGGGTCGCTTGGATGAAGCGTTTCATGGACAATGACAC
CCGTTATAGCACGTTCGCGTGTGAGAATCCGAACTCGACCgccGTgAGCGATTTTCGCACC
GCGAACTGTTCGTAAa

IV Supplementary References

(1) Martínez-García, E.; Aparicio, T.; Lorenzo, V. de; Nikel, P. I. New Transposon Tools Tailored for Metabolic Engineering of Gram-Negative Microbial Cell Factories. *Frontiers Bioeng Biotechnology* 2014, *2*, 46. https://doi.org/10.3389/fbioe.2014.00046.

(2) Franden, M. A.; Jayakody, L.; Li, W.-J.; Wagner, N. J.; Cleveland, N. S.; Michener, W. E.; Hauer, B.; Blank, L. M.; Wierckx, N.; Klebensberger, J.; Beckham, G. T. Engineering Pseudomonas Putida KT2440 for Efficient Ethylene Glycol Utilization. *Metab Eng* 2018, *48* (Biochim. Biophys. Acta 1435 153–9 1999), 197–207. https://doi.org/10.1016/j.ymben.2018.06.003.

(3) Mückschel, B.; Simon, O.; Klebensberger, J.; Graf, N.; Rosche, B.; Altenbuchner, J.; Pfannstiel, J.; Huber, A.; Hauer, B. Ethylene Glycol Metabolism by Pseudomonas Putida. *Appl Environ Microb* 2012, *78* (24), 8531--8539. https://doi.org/10.1128/aem.02062-12.

(4) Li, W.-J.; Jayakody, L. N.; Fr; en, M. A.; Wehrmann, M.; Daun, T.; Hauer, B.; Blank, L. M.; Beckham, G. T.; Klebensberger, J.; Wierckx, N. Laboratory Evolution Reveals the Metabolic and Regulatory Basis of Ethylene Glycol Metabolism by Pseudomonas Putida KT2440. *Environ Microbiol* 2019, *21* (10), 3669--3682. https://doi.org/10.1111/1462-2920.14703.

(5) Li, W.-J.; Narancic, T.; Kenny, S. T.; Niehoff, P.-J.; O'Connor, K.; Blank, L. M.; Wierckx, N. Unraveling 1,4-Butanediol Metabolism in Pseudomonas Putida KT2440. *Front Microbiol* 2020, *11*, 382. https://doi.org/10.3389/fmicb.2020.00382.

(6) Calero, P.; Jensen, S. I.; Nielsen, A. T. Broad-Host-Range ProUSER Vectors Enable Fast Characterization of Inducible Promoters and Optimization of p-Coumaric Acid Production in Pseudomonas Putida KT2440. *Acs Synth Biol* 2016, *5* (7), 741--753. https://doi.org/10.1021/acssynbio.6b00081.

(7) Martínez-García, E.; Aparicio, T.; Goñi-Moreno, A.; Fraile, S.; Lorenzo, V. de. SEVA 2.0: An Update of the Standard European Vector Architecture for de-/Re-Construction of Bacterial Functionalities. *Nucleic Acids Res* 2015, *43* (D1), D1183--D1189. https://doi.org/10.1093/nar/gku1114.

(8) Aparicio, T.; Lorenzo, V. de; Martínez-García, E. Improved Thermotolerance of Genome-Reduced Pseudomonas Putida EM42 Enables Effective Functioning of the PL/CI857 System. *Biotechnol J* 2019, *14* (1), 1800483. https://doi.org/10.1002/biot.201800483.

(9) Tozakidis, I. E. P.; Lüken, L. M.; Üffing, A.; Meyers, A.; Jose, J. Improving the Autotransporter-Based Surface Display of Enzymes in Pseudomonas Putida KT2440. *Microb Biotechnol* 2019, *0* (0), 176–184. https://doi.org/10.1111/1751-7915.13419.

(10) Cui, Y.; Chen, Y.; Liu, X.; Dong, S.; Tian, Y.; Qiao, Y.; Mitra, R.; Han, J.; Li, C.; Han, X.; Liu, W.; Chen, Q.; Wei, W.; Wang, X.; Du, W.; Tang, S.; Xiang, H.; Liu, H.; Liang, Y.; Houk, K. N.; Wu, B. Computational Redesign of a PETase for Plastic Biodegradation under Ambient Condition by the GRAPE Strategy. *Acs Catal* 2021, *11* (3), 1340–1350. https://doi.org/10.1021/acscatal.0c05126.
